# Telomerase gene therapy ameliorates the effects of neurodegeneration associated to short telomeres in mice

**DOI:** 10.18632/aging.101982

**Published:** 2019-05-28

**Authors:** Kurt Whittemore, Aksinya Derevyanko, Paula Martinez, Rosa Serrano, Martí Pumarola, Fàtima Bosch, Maria A. Blasco

**Affiliations:** 1 Telomeres and Telomerase Group, Molecular Oncology Program, Spanish National Cancer Centre (CNIO), Madrid28029, Spain; 2 Unit of Murine and Comparative Pathology (UPMiC), Department of Animal Medicine and Surgery, Veterinary Faculty, Universitat Autònoma de Barcelona, 08193 Bellaterra (Cerdanyola del Vallès), Barcelona, Spain; 3 Networking Research Center on Bioengineering, Biomaterials and Nanomedicine (CIBER-BBN), Universitat Autònoma de Barcelona, 08193 Bellaterra (Cerdanyola del Vallès), Barcelona, Spain; 4 Center of Animal Biotechnology and Gene Therapy, Department of Animal Medicine and Surgery, Universitat Autònoma de Barcelona, Bellaterra 08193, Spain; 5 Center of Animal Biotechnology and Gene Therapy, Department of Biochemistry and Molecular Biology, School of Veterinary Medicine, Universitat Autònoma de Barcelona, Bellaterra 08193, Spain

**Keywords:** telomerase, gene therapy, neurodegeneration, TERT

## Abstract

Neurodegenerative diseases associated with old age such as Alzheimer’s disease present major problems for society, and they currently have no cure. The telomere protective caps at the ends of chromosomes shorten with age, and when they become critically short, they can induce a persistent DNA damage response at chromosome ends, triggering secondary cellular responses such as cell death and cellular senescence. Mice and humans with very short telomeres owing to telomerase deficiencies have an earlier onset of pathologies associated with loss of the regenerative capacity of tissues. However, the effects of short telomeres in very low proliferative tissues such as the brain have not been thoroughly investigated. Here, we describe a mouse model of neurodegeneration owing to presence of short telomeres in the brain as the consequence of telomerase deficiency. Interestingly, we find similar signs of neurodegeneration in very old mice as the consequence of physiological mouse aging. Next, we demonstrate that delivery of telomerase gene therapy to the brain of these mice results in amelioration of some of these neurodegeneration phenotypes. These findings suggest that short telomeres contribute to neurodegeneration diseases with aging and that telomerase activation may have a therapeutic value in these diseases.

## Introduction

Parkinson’s, are associated with aging, and their pre-valence is increasing as there are more individuals that reach older ages [1–3]. To date, there are no curative treatments for any of these diseases owing to the fact that their molecular origins are still poorly understood. Instead, palliative treatments are directed to alleviate downstream events, such as beta-amyloid deposition in the case of Alzheimer’s or lack of dopamine generation in the case of Parkinson’s due to the loss of dopaminergic neurons.

Telomeres are heterochromatic protective structures at the ends of chromosomes that consist of TTAGGG repeats bound by a six-protein complex known as shelterin [4,5]. A minimum length of telomeric repeats is necessary for shelterin binding and protection [4,5]. Telomerase is a reverse transcriptase which can elongate telomeres *de novo* by the addition of telomeric repeats onto chromosome ends [6], in this manner compensating progressive telomere attrition as a consequence of cell division. Telomerase is composed of two essential subunits, the telomerase reverse transcriptase (TERT) and the telomerase RNA component (Terc), which is used as a template for the synthesis of telomeric repeats [6]. Adult tissues, including the stem cell compartments, do not have sufficient telomerase activity to compensate for telomere shortening associated with tissue regeneration and cell division [7–9]. When telomeres reach a critically short length, this triggers activation of a persistent DNA damage response at telomeres and the subsequent induction of cellular senescence or apoptosis [5,10]. Short/dysfunctional telomeres are considered one of the primary hallmarks of aging both in mice and humans, as they lead to other hallmarks of aging, such as genomic instability, cellular senescence, and loss of the regenerative capacity of tissues [11]. In particular, critically short telomeres in the stem cell compartments lead to impaired tissue renewal and homeostasis [12–14]. Interestingly, the rate of telomere shortening throughout lifespan is influenced by both genetic factors (i.e. mutations in genes necessary for telomere maintenance) and environmental factors (i.e. cigarette smoke has a negative effect) [15,16]. Interestingly, there are a number of diseases associated to mutations in telomerase and other telomere maintenance genes known as “telomere syndromes”, which include dyskeratosis congenita, aplastic anemia and pulmonary fibrosis, among others (for a review see [17]). These syndromes are characterized by the presence of extremely short telomeres, which prematurely impair the regenerative capacity of tissues, affecting both high and low proliferative tissues [17,18]. Prior to the discovery of human “telomere syndromes”, similar findings were made by studying mice genetically modified to lack the telomerase RNA component (*Terc^-/-^*) [19]. *Terc*-deficient mice have shorter telomeres with increasing mouse generations and this results in a progressive decrease of both median and maximum longevity [20,21]. These mice show pre-mature appearance of different pathologies affecting both proliferative and non-proliferative tissues, which are accompanied by an impaired regenerative capacity (reviewed in [10,13]).

In support of critically short telomeres being a determinant of aging and longevity, we previously showed that increased TERT expression in the context of cancer resistant transgenic mice was sufficient to delay aging and extend mouse longevity by 40% [22]. We further demonstrated that telomerase reactivation in adult tissues by using adeno-associated viruses (AAV9-*Tert*) was able to significantly delay age-related diseases and increase longevity in wild-type mice [23]. In particular, AAV9-*Tert* treatment resulted in reduced age-related osteoporosis, reduced glucose intolerance, increased neuromuscular coordination, enhanced memory in an object recognition test, improved mitochondrial fitness, and delayed cancer, thus demonstrating that telomere shortening is causative of aging and is at the origin of a wide range of age-associated diseases, including cognitive decline [23]. More recently, we have also shown that AAV9-mediated telomerase activation has therapeutic effects in pre-clinical mouse models for diseases associated with short telomeres such as aplastic anemia [24], myocardial infarction [25], and pulmonary fibrosis [26].

In spite of the fact that the brain is a low-proliferative tissue, there are regenerative areas within the brain such as the hippocampus, the subventricular zone (SVZ), and the olfactory bulb. Interestingly, several studies suggest the presence of short telomeres in patients with advanced Alzheimer’s disease [27–32]. In the case of Parkinson’s disease, extensive research has not been performed. A few studies have found a correlation between short telomere length and Parkinson’s disease [33,34], while other studies found no correlation [35,36]. These studies were performed with peripheral blood leukocytes, and telomere length was not measured in the brain cells implicated in Parkinson’s disease. In mice, we have previously shown that telomere attrition in the context of the *Terc*-deficient mouse model [19] impairs *in vitro* proliferation of adult neural stem cells from the SVZ [37], and that telomere shortening in mice disrupts neuronal differentiation and neurogenesis [38]. These findings pose the interesting hypothesis that telomerase reactivation in the brain may have significant therapeutic effects. Indeed, both in our AAV9-*Tert* mediated telomerase reactivation model [23] and in a genetic *Tert* reactivation mouse model [39], there were beneficial effects associated to telomerase reactivation in the brain. In particular, telomerase re-expression in a telomerase-deficient mouse model with short telomeres, resulted in a larger brain weight, a thicker myelin sheath, better performance in an innate avoidance test as a measure of the health of the olfactory bulb, and increased molecular markers of Ki67, Sox2, doublecortin, and Olig2 [39]. Similarly, telomerase re-expression in the brain using AAV9 gene therapy in adult wild-type mice was sufficient to improve cognitive function [23].

Here, we set to evaluate whether mice with short telomeres in the brain owing to telomerase deficiency could be used as a *bona fide* neurodegeneration model. To this end, we characterized the molecular consequences of the presence of short telomeres in the neurogenic areas of the brain as well as the potential cognitive defects associated to short telomeres in these mice. We also explored whether similar signs of neurodegeneration can be found in wild-type mice associated to physiological mouse aging. Finally, we demonstrate that telomerase activation using AAV9-*Tert* gene therapy can ameliorate the brain phenotypes in these mice**.**

## RESULTS

### Histological and molecular defects associated to shorter telomeres in the brain of telomerase-deficient mice

**Figure 1:**
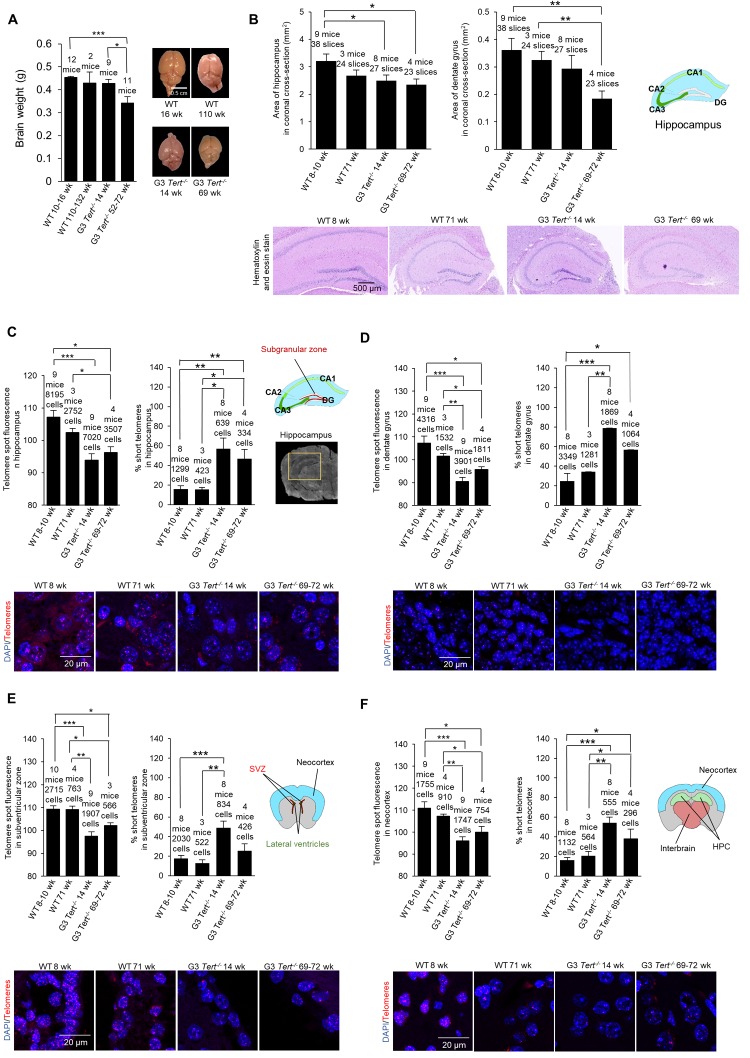
**Mice deficient for telomerase have smaller brains, shorter telomeres, more proliferation, more DNA damage, and less neurogenesis. **(**A**) Brain weight and representative images of young and old wild-type and G3 *Tert^-/-^* mice. (**B**) Area of hippocampus and dentate gyrus in untreated mice quantified from representative images of brain sections stained with hematoxylin and eosin. (**C**) Q-FISH for telomere spot fluorescence measured in the hippocampus, (**D**) the dentate gyrus specifically, (**E**) the subventricular zone, and (**F**) the neocortex. The mean telomere spot fluorescence is shown. The percentage of short telomeres is also shown with “short” being defined as a fluorescence intensity less than the 15^th^ percentile of the fluorescence intensity values of a control sample. Cartoon diagrams label the different regions of the brain. In part (**C**), A scan of a coronal brain cross-section without fluorescence is shown with the hippocampus region highlighted in yellow. Representative images show the telomere spots labeled with Cy3-Tel probe (in red), and nuclei stained with DAPI (blue).

First, we set to characterize brain phenotypes in the telomerase-deficient *Tert^-/-^* mouse model used in this study [40–43]. We first weighed the brains of age-matched wild-type and third generation (G3) *Tert^-/-^* mice, which have shorter telomeres owing to telomerase deficiency for three generations. We observed that the brains of G3 *Tert^-/-^* mice showed a tendency to be smaller than those of age-matched wild-type controls (Figure 1A), in agreement with a previous report [39]. A reduced brain size may be indicative of neuro-degeneration as this phenotype is also observed in patients with advanced Alzheimer’s disease [44]. In this regard, we quantified the size of the hippocampus and dentate gyrus (DG) of the different mouse cohorts (Figure 1B). We found that G3 *Tert^-/-^* mice also show smaller hippocampus and dentate gyrus regions than wild-type mice (Figure 1B).

We next set to characterize the molecular events associated to telomerase deficiency in the brain of G3 *Tert^-/-^* mice. We first determined telomere length in several brain regions of age-matched wild-type and G3 *Tert^-/-^* mice, including the hippocampus since this structure is critical for learning, memory, memory for episodic events, and neurogenesis [45,46], as well as the DG area within the hippocampus since this region is involved in neurogenesis [47–51] (see scheme in Figure 1C). In addition, we also studied telomere length in the subventricular zone which is important for neurogenesis in the adult brain [52], and the neocortex which is important for higher-order brain functions such as sensory perception, cognition, generation of motor commands, spatial reasoning, and language [53]. To this end, we performed quantitative telomere FISH (Q-FISH) directly on coronal paraffin brain sections from young wild-type (8-10-weeks old), old wild-type (71-weeks old), young G3 *Tert^-/-^* (14-weeks old), and old G3 *Tert^-/-^* mice (69-72-weeks old). We found that telomeres were significantly shorter in the hippocampus, dentate gyrus, subventricular zone, and neocortex of G3 *Tert^-/-^* mice compared to the same regions in wild-type mice (Figure 1C-F). Accordingly, we also found that the percentage of short telomeres in G3 *Tert^-/-^* in these brain regions was higher (Figure 1C-F). Short telomeres were defined as telomeres with a fluorescence intensity less than the 15th percentile of the intensity values of a control.

**Figure 2:**
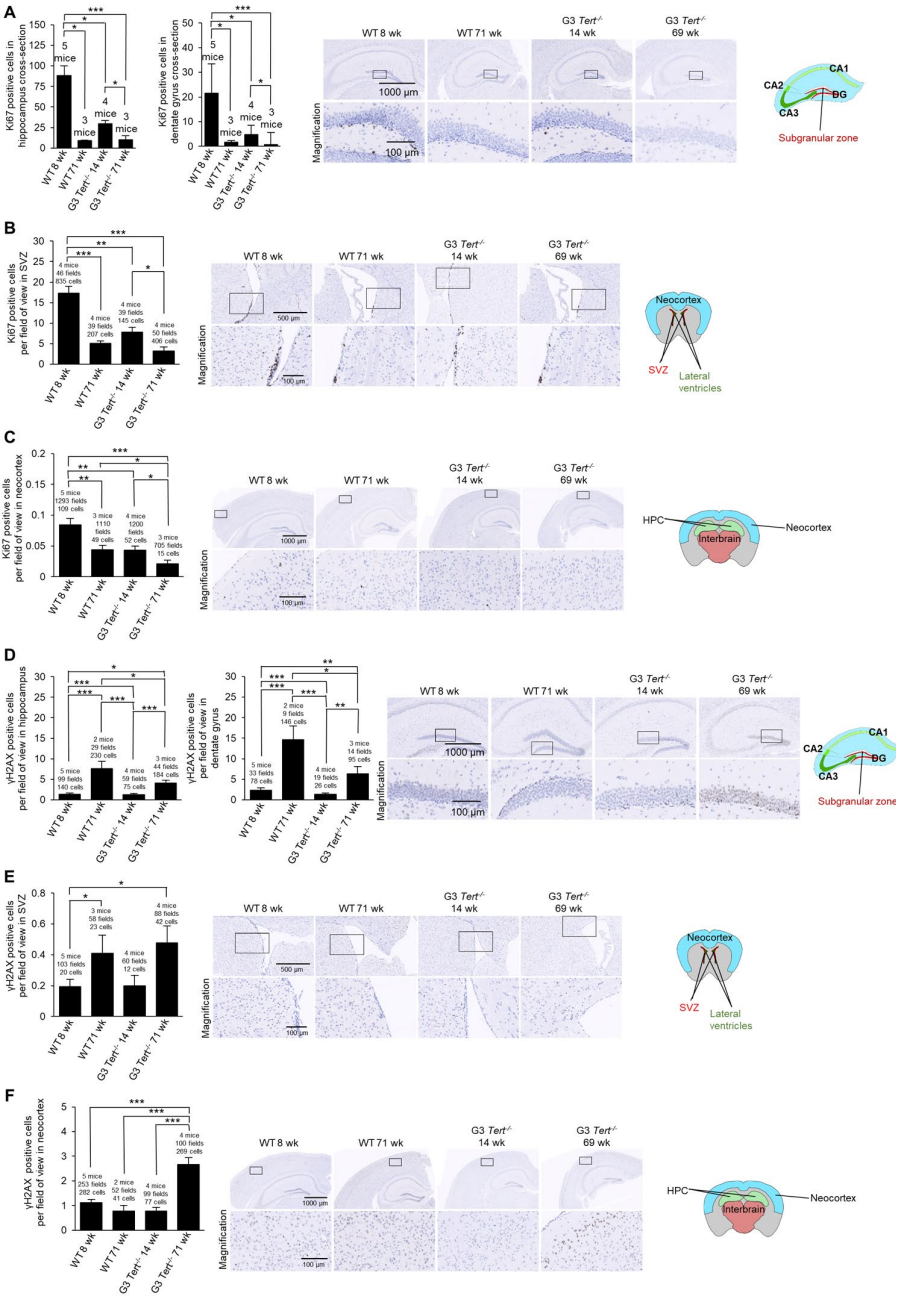
**Immunohistochemistry of Ki67 and γH2AX in the brain**. (**A-F**) The quantification and representative images of the immunohistochemistry for positive cells per field of view for (**A-C**) Ki67, and (**D-F**) γH2AX in brain regions such as the hippocampus, dentate gyrus, subventricular zone (SVZ) of the lateral ventricle anterior to the hippocampus level, and the neocortex. The data is shown for young and old wild-type and G3 *Tert^-/-^* mice. Data represent the mean ±SE of analyzed mice within each group. For the histopathology results, the number of mice analyzed per group is indicated, as well as the number of fields of view, and the number of positive cells. The *t*-test was used for statistical analysis. *p<0.05; **p<0.01; ***p<0.001.

Next, we determined whether shorter telomeres in these brain regions of *Tert^-/-^* mice were associated to reduced proliferation and increased DNA damage [54]. To this end, we used immunohistochemistry to determine the number of Ki67-positive cells as a marker of cycling cells and the number of cells positive for γH2AX as a marker for DNA damage. In the case of wild-type mice, we observed significantly fewer Ki67-positive cells in older mice (71-weeks old) compared to younger ages (8-weeks old) in the hippocampus, the dentate gyrus, subventricular zone (SVZ) of the lateral ventricle anterior to the hippocampus level, and the neocortex (Figure 2A-C). Interestingly, young G3 *Tert^-/-^* mice (14-weeks old) also showed lower numbers of Ki67-positive cells than age-matched wild-type controls in these brain regions, and this was further reduced in the old G3 *Tert^-/-^* mice (Figure 2A-C). Regarding DNA damage, we found increased numbers of γH2AX-positive cells in the hippocampus, dentate gyrus, and SVZ of old wild-type mice (71-weeks old) compared to young mice (8-weeks old) (Figure 2D,E). Young G3 *Tert^-/-^* mice also showed significantly lower numbers of γH2AX-positive cells in the hippocampus, dentate gyrus, and subventricular zone compared to older G3 *Tert^-/-^* mice (Figure 2D,E). In the neocortex, more γH2AX associated DNA damage was only observed in the old G3 *Tert^-/-^* mice (Figure 2F). These results indicate that physiological aging in wild-type mice, as well as accelerated telomere shortening as a consequence of telomerase-deficiency in the *Tert^-/-^* mice, lead to decreased proliferation and increased DNA damage in the hippocampus, dentate gyrus, SVZ, and neocortex.

**Figure 3:**
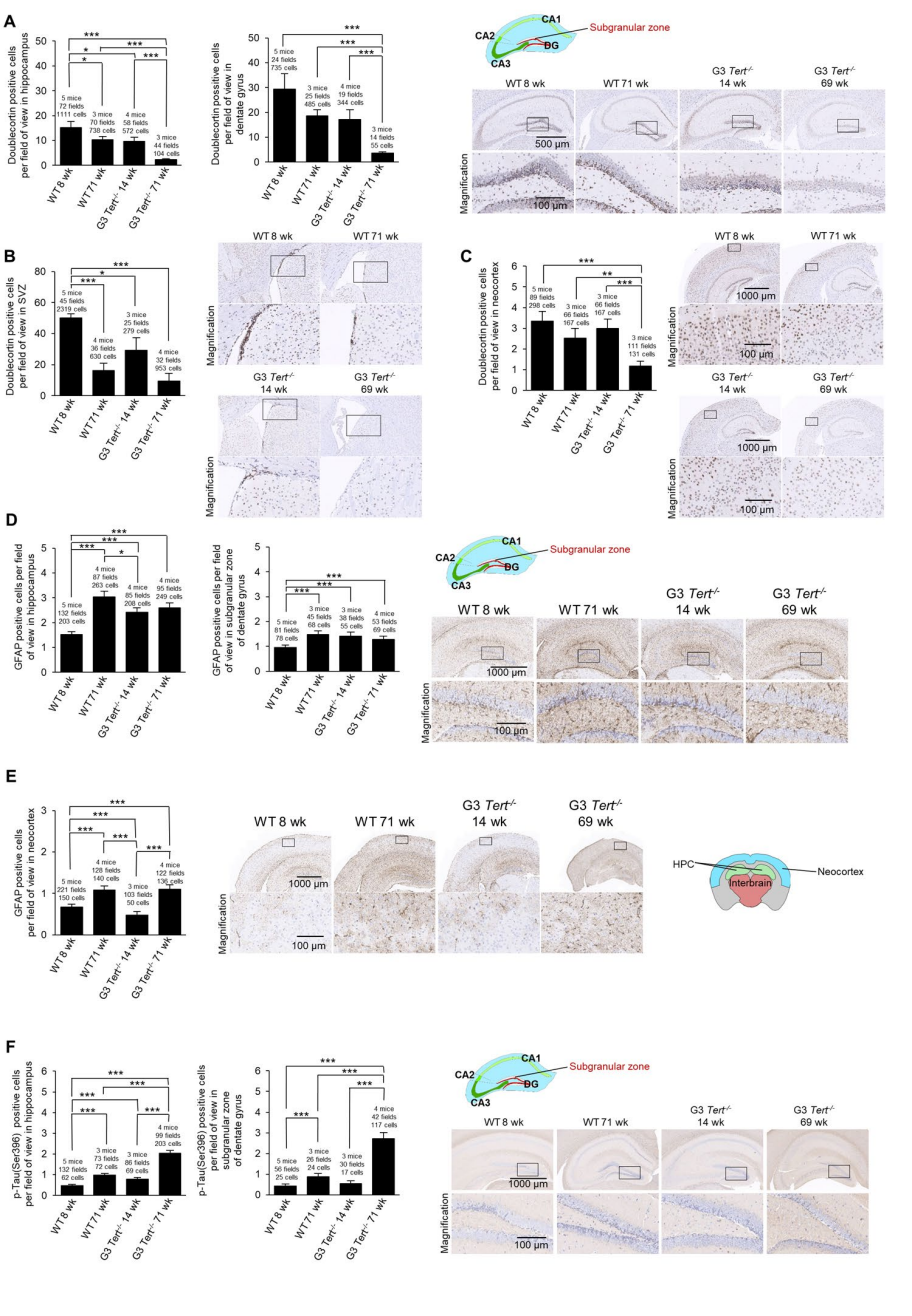
**Immunohistochemistry of GFAP and Tau in the brain**. (**A-F**) The quantification and representative images of the immunohistochemistry for positive cells per field of view for (**A-C**) doublecortin, (**D-E**) glial fibrillary acidic protein (GFAP), and (**F**) p-Tau(Ser396) in brain regions such as the hippocampus, dentate gyrus, and the neocortex. The data is shown for young and old wild-type and G3 *Tert^-/-^* mice. Data represent the mean ±SE of analyzed mice within each group. For the histopathology results, the number of mice analyzed per group is indicated, as well as the number of fields of view, and the number of positive cells. The *t*-test was used for statistical analysis. *p<0.05; **p<0.01; ***p<0.001.

Next, we set to address the impact of shorter telomeres on neurogenesis, inflammation, and formation of tau protein aggregations. In this regard, neurogenesis has been suggested to act as a brain repair mechanism which could mitigate the effects of neurodegeneration that occurs with Alzheimer’s disease and possibly aging [54,55]. Doublecortin is expressed in developing neurons and it is considered a *bona fide* marker of neurogenesis [39,56]. To this end, we set to study the expression of doublecortin in various brain regions of both wild-type and G3 *Tert^-/-^* mice. We found that the number of cells and G3 *Tert^-/-^* mice. We found that the number of cells expressing doublecortin was significantly decreased in expressing doublecortin was significantly decreased in old wild-type mice (71-weeks old) compared to young (8-weeks old) wild-type mice in the hippocampus and the SVZ, and showed the same tendency in the dentate gyrus and the neocortex (Figure 3A-C). Young G3 *Tert^-/-^* mice also showed lower numbers of cells ex-pressing doublecortin in the hippocampus, dentate gyrus, subventricular zone, and neocortex compared to age-matched wild-type controls, and this was further aggravated with increasing age (Figure 3A-C). The same trends were observed for doublecortin expression in the parietotemporal region of the cerebral cortex (Supplementary Figure S1A), and the occipital region of the cerebral cortex (Supplementary Figure S1B). Neurogenic niches were also identified in hematoxylin and eosin stains in the parietal subventricular zone area, and the young wild-type mice had higher levels of neurogenic niches than old wild-type mice (Supplementary Figure S1C). In agreement with this finding, young G3 *Tert^-/-^* mice had more neurogenic niches than old G3 *Tert^-/-^* mice, but less than young wild-type mice (Supplementary Figure S1C). These findings indicate that shorter telomeres associated with aging and to telomerase-deficiency correlate with impaired neurogenesis in different regions of the brain.

A sign of brain aging is neuroinflammation [57]. In particular, the expression of glial fibrillary acidic protein (GFAP) by astrocytes increases with aging as astrogliosis and neuroinflammation occurs, and such an increase is also observed in mouse models with in-creased neuroinflammation [58–60]. The increase in GFAP expression accompanies increased expression of inflammatory cytokines, accumulation of proteotoxic aggregates, and senescence [58]. To address, whether physiological aging and/or short telomeres in the context of telomerase deficiency lead to increased inflammation in the brain, we measured the number of immune astrocyte cells with strong expression of the GFAP marker [61]. We observed that young wild-type mice have low levels of GFAP in the hippocampus and dentate gyrus, whereas old wild-type, young G3 *Tert^-/-^*, and old G3 *Tert^-/-^* mice have more GFAP-positive astrocytes in these areas (Figure 3D). We did not find significantly more GFAP in the neocortex of G3 *Tert^-/-^* mice compared to wild-type, but did find an increase of GFAP with age in both generations (Figure 3E).

Another sign of brain aging is the accumulation of tau or abnormal phosphorylation of tau protein located throughout the brain in various cell types such as neurons, astrocytes, and oligodendrocytes, ultimately resulting in aggregates and neurofibrillary tangles [62–64] . Indeed, tau has been associated with Alzheimer’s because hyperphosphorylation of tau results in loss of its biological activity [65]. To address this in our mouse models, we measured protein aggregation by determining the number of cells positive for tau phosphorylated at serine 396 [66,67]. We observed more cells positive for phosphorylated tau in the hippocampus and dentate gyrus of old wild-type mice, and this effect was further increased in old G3 *Tert^-/-^* mice (Figure 3F).

In summary, these findings indicate that short telomeres in the brain contribute to impair brain neurogenesis and to increase neuroinflammation and abnormal tau phosphorylation. Interestingly, similar phenotypes were observed associated to physiological aging in very old wild-type mice.

### Telomerase-deficient mice are more susceptible to MPTP neurotoxin

To determine whether mice deficient for telomerase were more susceptible to cellular damage, we per-formed an experiment with the MPTP (1-methyl-4-phenyl-1,2,3,6-tetrahydropyridine) neurotoxin. MPTP specifically damages dopaminergic neurons in the substantia nigra region of the brain [68]. Inside these neurons, MPTP is metabolized to MPP+ which then interferes with complex I of the electron transport chain in mitochondria, ultimately resulting in oxidative damage. The MPTP neurotoxin model is a common model used to study Parkinson’s disease [69]. In our experiment, 12-16-week old male wild-type and G3 *Tert^-/-^* mice were injected IP (intraperitoneally) with 0.5, 5, 10, or 20 mg/kg MPTP and sacrificed 7 days later (scheme in Supplementary Figure S2A). An additional injection was administered 2 hours later since multiple injections have been suggested by previous protocols [69]. A footprint test resulted in a trend showing that wild-type and G3 *Tert^-/-^* mice injected with the MPTP had a shorter hind paw stride length (Supplementary Figure S2B). In addition, we performed a tail suspension test to measure mobility. In particular, we hung the mice from the tail for a period of 5 min and measured their immobility time. Mice that are old, unhealthy, or more depressed, move less in the tail suspension test [70]. We found that wild-type mice exhibited a shorter immobility time than MPTP-treated wild-type and G3 *Tert^-/-^* mice (Supplementary Figure S2C). After 7 days, the mice were sacrificed, and the brain was preserved with formalin. In order to investigate the dopaminergic neurons, we performed an immuno-fluorescence experiment for the tyrosine hydroxylase marker. Tyrosine hydroxylase is important in dopaminergic neurons since it converts the amino acid L-tyrosine to L-DOPA which is a precursor for dopamine [71]. We found that 12-16-week old male mice injected with higher doses of MPTP had lower levels of tyrosine hydroxylase (Figure S2D). Interestingly, saline-treated control G3 *Tert^-/-^* mice showed less tyrosine hydroxylase than wild-type mice (Figure S2D), and G3 *Tert^-/-^* mice treated with a low dose of MPTP showed less styrosine hydroxylase than similarly treated wild-type mice (Figure S2D).

Additionally, old wild-type and old G3 *Tert^-/-^* mice had lower levels of tyrosine hydroxylase than young mice (Supplementary Figure S2E). Together these results suggest that G3 *Tert^-/-^* mice are more susceptible to damage to dopaminergic neurons in the substantia nigra due to MPTP neurotoxin, and this damage results in decreased mobility.

### Behavioral defects associated to shorter telomeres in the brain of telomerase-deficient mice

We previously showed that old wild-type mice perform more poorly than young mice in tests such as the object recognition test, rotarod test, and tightrope test [23], suggesting a negative impact of telomere shortening associated with aging in these tests. Thus, we here set to address whether telomerase deficient mice performed more poorly than age-matched wild-type controls in different behavioral and cognitive tests. The tightrope test measures the ability of mice to balance on a tightrope, and the rotarod test measures neuromuscular coordination while running on a rotating rod. In addition, we performed a tail suspension test to measure mobility as described previously in this text. We found that late generation G1, G2, G3, and G4 *Tert^-/-^* mice performed worse than age-matched wild-type mice in the tightrope test and rotarod test at older ages (Supplementary Figure S3A,B). Furthermore, we found that older G2 *Tert^-/-^* mice are immobile in the tail suspension test for longer periods of time than age-matched wild-type mice and this was further aggravated in the G3 and G4 *Tert^-/-^* mice (Supplementary Figure S3C), suggesting a negative impact of progressively shorter telomeres in this test. Thus, telomerase deficiency and shorter telomeres lead to significantly impaired performance in several tests that measure neuromuscular coordination and balance.

To further assess a potential impact of telomerase deficiency on cognitive function, we subjected the different mouse cohorts to an object recognition test. We found that G4 *Tert*^-/-^ mice spent less time investigating a novel object in their environment than age-matched wild-type mice (Supplementary Figure S3D), which is an indication of poor memory in G4 *Tert*^-/-^ mice with shorter telomeres [72]. Together, these findings indicate that mice with shorter telomeres show noticeable behavioral phenotypes consisting of a poorer performance in behavioral and cognitive tests.

Finally, we set to analyze the brain metabolic activity in the different mouse cohorts as another indication of brain defects in these mice. To this end, we compared differences in metabolic activity in the brains of wild-type and G3 *Tert^-/-^* mice by using positron emission tomography (PET) to detect fluorodeoxyglucose (FDG) in the brain after injection. Brain glucose metabolism has been found to decrease in human patients with Alzheimer’s disease and during senile dementia [73–75]. We found that G3 *Tert^-/-^* mice had a lower standard glucose uptake value (SUV) in the brain compared to wild-type brains, which is an indication of lower metabolic activity (Supplementary Figure S3E). The weights of the mice were taken into account when calculating the SUV. Thus, telomerase deficiency and shorter telomeres resulted in decreased glucose metabolism in the brain, which is associated with poorer cognitive performance and increased neurodegeneration.

Together, these findings show that *Tert^-/-^* mice with short telomeres exhibit more neurodegeneration than wild-type mice, suggesting an impact of telomere length on brain aging in mice.

### AAV9-mediated telomerase transduction of mouse brains

**Figure 4:**
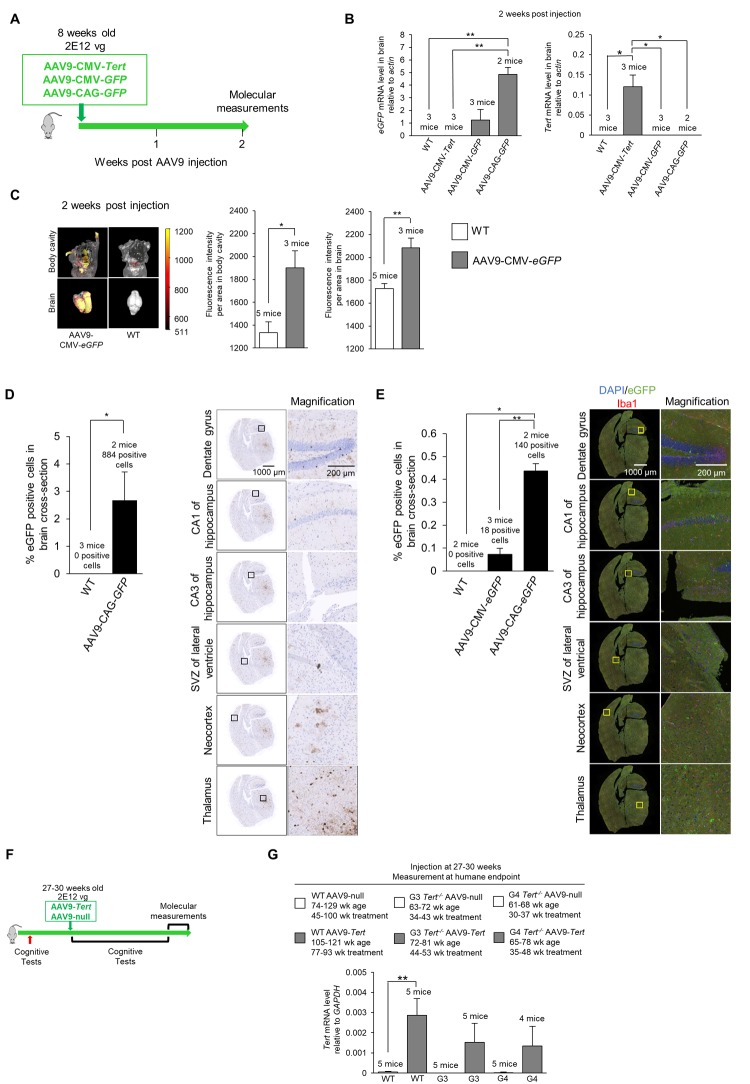
**Experiment scheme and confirmation of virus transduction in the brain**. (**A**) Scheme of the gene therapy experiment. Wild-type mice (8-week old) were injected IV with AAV9-CMV-*Tert*, AAV9-CMV-*eGFP*, or AAV9-CAG-*eGFP*, and sacrificed 2 weeks after injection. (**B**) Level of *eGFP* and *Tert* mRNA relative to *actin* in the brain as measured by qPCR 2 weeks post IV tail injection with 2E12 vg of AAV9-CMV-*Tert*, AAV9-CMV-*eGFP*, AAV9-CAG-*eGFP*, or no virus. (**C**) Quantification and representative images of fluorescence as measured by an IVIS instrument in the body and brain of wild-type mice and mice injected IV in the tail with AAV9-CMV-*eGFP*. (**D**) Quantification and representative images of eGFP positive cells in the brain as measured by immunohistochemistry in mice injected in the tail with 2E12 vg AAV9-CAG-*eGFP* or no virus. The percentage of eGFP positive cells was calculated from the whole coronal brain cross-section. The representative images show multiple regions throughout the brain as labelled. (**E**) Quantification and representative images of eGFP positive cells in the brain as measured by immunofluorescence in mice injected IV in the tail with 2E12 vg AAV9-CMV-*eGFP*, AAV9-CAG-*eGFP*, or no virus. The percentage of eGFP positive cells was calculated from the whole coronal brain cross-section. The representative images show multiple regions throughout the brain as labelled with DAPI stained nuclei in blue, eGFP in green, and Iba1 for microglia in red. (**F**) Scheme of experiment with injection of AAV9-*Tert* into young mice. The mice (wild-type, G3 *Tert^-/-^*, and G4 *Tert^-/-^* mice) were treated at a young age (27-30 weeks) by the IV tail injection of 2E12 vg of AAV9-*Tert* or AAV9-null virus. (**G**) The mRNA level of *Tert* in the brain at the humane endpoint. The mRNA level was measured by qPCR relative to *GAPDH*. The ages of the mice as well as the number of weeks of treatment for each group are indicated above the graph. Data represent the mean ±SE of analyzed mice within each group. The number of mice analyzed per group is indicated. The *t*-test was used for statistical analysis. *p<0.05; **p<0.01.

Next, we set to study whether expression of telomerase in these mouse models could ameliorate the signs of neurodegeneration described above. Previous reports have shown that adeno associated viruses of the serotype 9 (AAV9) are able to cross the blood brain barrier and transduce a significant percentage of cells [76–84]. To verify whether in our experimental setting an IV (intravenous) injection of the AAV9 vector could cause effective expression of the *Tert* transgene in the brain, we injected 8-week old mice with the AAV9-CMV-*Tert*, AAV9-CMV-*eGFP*, or AAV9-CAG-*eGFP* vector, and the mice were sacrificed after 2 weeks of treatment to study expression of the *eGFP* or *Tert* transgenes (scheme in Figure 4A). The “CMV” abbreviation corresponds to the cytomegalovirus promoter, and the “CAG” abbreviation corresponds to the high-expression synthetic CAG promoter [85]. We first determined whether AAV9 carrying either *eGFP* or *Tert* genes caused gene expression in the mouse brain upon intravenous (IV) injection in the tail with a dose of 2E12 viral genomes (vg) per mouse (see Methods). To this end, 8-week old mice were injected with AAV9 virus particles containing either *Tert* or *eGFP* (Figure 4A) and under either the CMV promoter [23–25], or the CAG promoter. Mice treated with the different viral vectors were sacrificed 2 weeks after tail injection and several different analyses were performed. We performed quantitative qPCR to detect *Tert* and *eGFP* mRNA expression in the brain (Figure 4B). We found expression of the *eGFP* gene in the brains of AAV9-*eGFP* treated mice but not in brains from the AAV9-*Tert* treated mice (Figure 4B). In turn, the *Tert* mRNA was only detected in the brain of AAV9-*Tert* treated mice but not in those treated with AAV9-*eGFP* (Figure 4B). We also measured eGFP fluorescence in the whole mouse, including the brain, using an IVIS instrument (Figure 4C). AAV9-*eGFP* treated mice displayed detectable eGFP fluorescence in the body as well as in the brain, whereas untreated wild-type control mice only displayed background levels of fluorescence (Figure 4C). Thus, these results confirmed that AAV9 viruses carrying either the *eGFP* or *Tert* genes were able to cross the blood-brain barrier and infect cells in the brain.

Note that the AAV9-CMV-*eGFP* virus should infect the same number of cells as the AAV9-CAG-*eGFP* since the viral capsid is the same. However, the CMV promoter results in lower levels of expression, and therefore we are unable to detect the AAV9-CMV-*eGFP* infected cells by immunohistochemistry or immunofluorescence. Therefore, the AAV9-CAG-*eGFP* vector was utilized to locate infected cells and determine the number of cells that were infected. We could not use an AAV9-CAG-*Tert* vector because the genetic material is too large to efficiently pack into the viral vector.

Next, in order to visualize eGFP expression in different brain areas by immunohistochemistry and immuno-fluorescence, we used the AAV9-CAG-*eGFP* vector, previously shown to have a very high expression level in the brain ([86,87]; see also Figure 4C). Upon tail injection, upon tail injection of AAV9-CAG-*eGFP* vectors, we used immunohistochemistry to detect *eGFP* positive cells throughout the entire brain, including the dentate gyrus, CA1 of the hippocampus, CA3 of the hippocampus, subventricular zone (SVZ) of the lateral ventricle, the neocortex, and the thalamus, which showed the highest eGFP expression (Figure 4D). Transduction of brain cells was also confirmed by immunofluorescence using an antibody against GFP, including all the brain areas studied (Figure 4E). We observed that the transduction efficiency of all cells in a whole brain cross-section from a position in the brain at the hippocampus level was approximately 2.5% as detected by immunohistochemistry (Figure 4D) and approximately 0.4% as detected by immunofluorescence (Figure 4E). This provides a range for the transduction efficiency which is in approximately the same range reported by other studies using different promoters [77,88]. We also addressed whether AAV9 transduced microglia which express the marker Iba1. However, we did not detect significant eGFP expression in the Iba1-positive microglia cells, also in agreement with previous reports [77,89].

Next, to address whether telomerase over-expression in the adult brain of mice could ameliorate signs of brain damage and neurodegeneration in mice, age-matched (27-30-weeks of age) wild-type, *G3 Tert^-/-^*, and G4 *Tert^-/-^* mice were treated with an IV tail injection of 2E12 vg of AAV9-*Tert* or AAV9-null vectors (scheme in Figure 4F). The mice were then followed throughout their lifespan until they reached the humane endpoint at which time we performed a number of molecular determinations. First, and in agreement with transduction of mouse brain, qPCR analysis demonstrated increased *Tert* mRNA expression in the brains of mice transduced with the AAV9-*Tert* vectors compared to those transduced with the null-vectors at the time of death (Figure 4G). Thus, increased *Tert* expression was maintained throughout the lifespan of the mice even until the humane endpoint. A graph of the body weights of the mice is presented in Supplementary Figure S4.

### AAV9-*Tert* gene therapy results in less signs of molecular aging in the brain in mice

**Figure 5:**
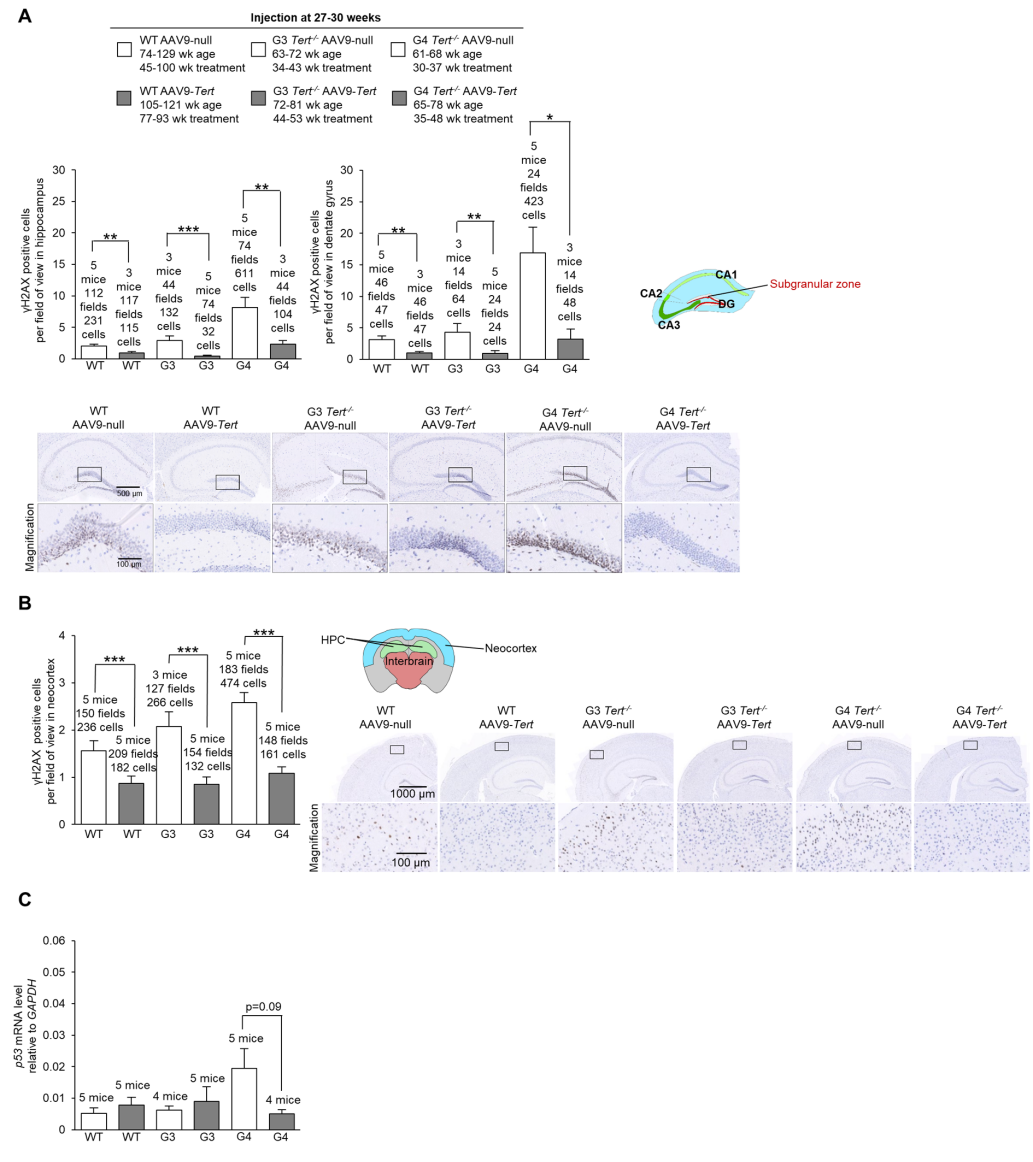
**Treatment with AAV9-*Tert* results in less DNA damage in the brain**. (**A-B**) Quantification and representative images of the histopathology for γH2AX in the (**A**) hippocampus, (**A**) dentate gyrus, and (**B**) neocortex for the cohort of mice (wild-type, G3 *Tert^-/-^*, and G4 *Tert^-/-^* mice) injected with 2E12 vg of AAV9-*Tert* or AAV9-null. (**C**) The level of *Trp53* mRNA in the brain as measured by qPCR relative to *GAPDH* at the humane endpoint. Data represent the mean ±SE of analyzed mice within each group. The number of mice analyzed per group is indicated. The *t*-test was used for statistical analysis. *p<0.05; **p<0.01; ***p<0.001.

**Figure 6:**
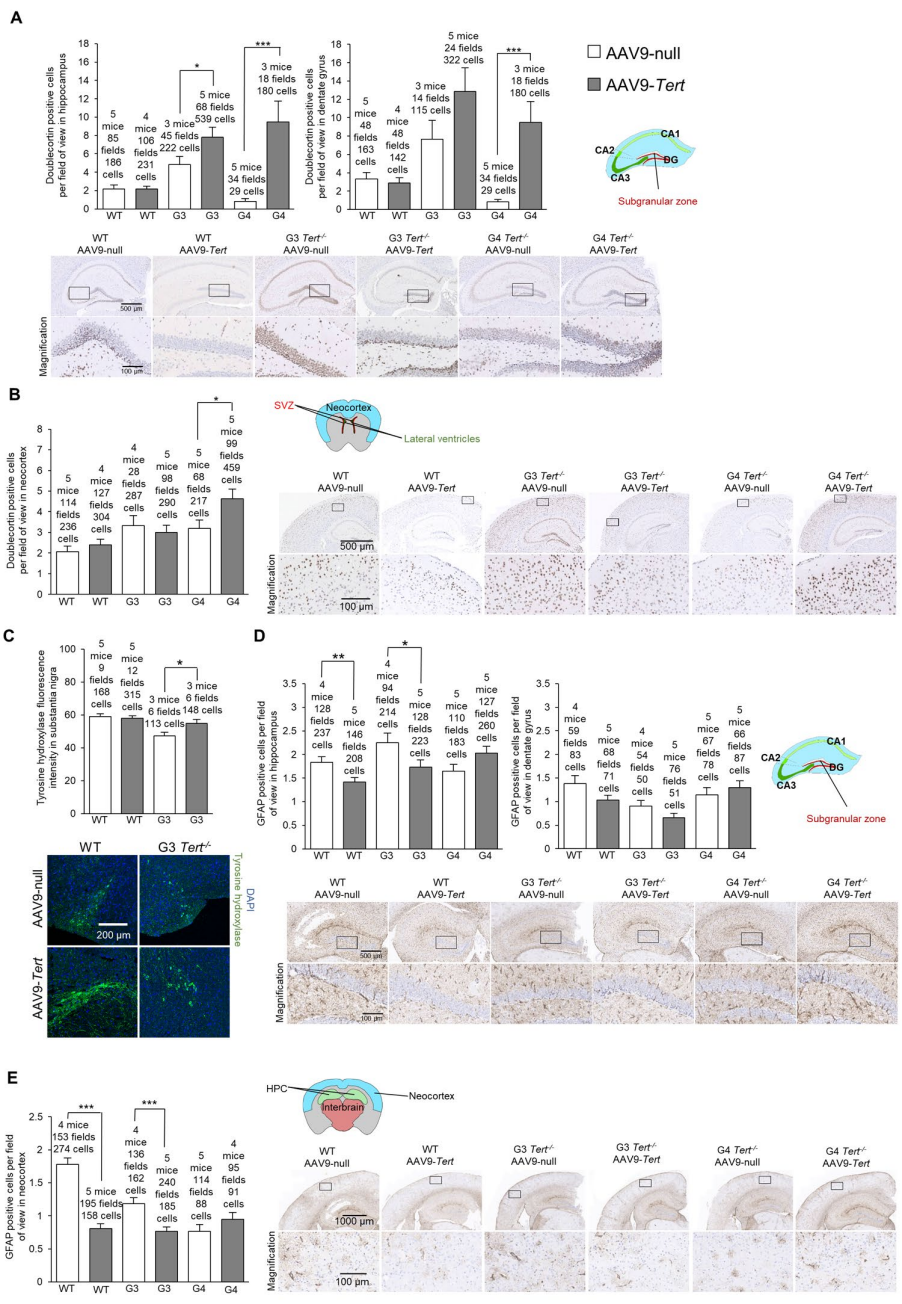
**Treatment with AAV9-*Tert* results in more neurogenesis and less inflammation in the brain**. (**A-B**) Quantification and representative images of the histopathology for doublecortin in the hippocampus (**A**), dentate gyrus (**A**), and neocortex (**B**). (**C**) Immunofluorescence of tyrosine hydroxylase in the substantia nigra. (**D-E**) Quantification and representative images of the histopathology for glial fibrillary acidic protein (GFAP) in the hippocampus (**D**), dentate gyrus (**D**), and (**E**) neocortex. Data represent the mean ±SE of analyzed mice within each group. The number of mice analyzed per group is indicated. The *t*-test was used for statistical analysis. *p<0.05; **p<0.01; ***p<0.001.

First, we assessed the presence of DNA damage at the humane endpoint using the γH2AX marker. We confirmed more cells with DNA damage with increasing *Tert^-/-^* generations compared to wild-type mice in the hippocampus, the dentate gyrus, and the neocortex of mice treated with AAV9-*null* vectors (Figure 5A,B). Interestingly, we observed significantly lower numbers of cells with DNA damage for wild-type, G3 *Tert^-/-^*, and G4 *Tert^-/-^* mice groups treated with AAV9-*Tert* in the hippocampus, dentate gyrus, and neocortex compared to the controls treated with the AAV9-null vectors (Figure 5A,B). We also observed reduced levels of the senescence and DNA damage marker *Trp53* mRNA in the AAV9-*Tert* treated G4 *Tert^-/-^* mice compared to the AAV9-null treated controls (Figure 5C), again suggesting less DNA damage in the telomerase-treated brains. We also addressed whether telomerase treatment had any impact on the regenerative neurons of the brain by quantifying cells positive for the neurogenesis marker doublecortin (Figure 6A,B). As expected, in the AAV9-null treated controls, we found significantly lower numbers of cells positive for doublecortin in the hippocampus and dentate gyrus, and of G4 *Tert^-/-^* mice compared to the wild-type mice (Figure 6A,B). Interestingly, AAV9-*Tert* treatment resulted in significantly increased numbers of doublecortin-positive cells in the hippocampus, the dentate gyrus, and the neocortex in G4 *Tert^-/-^* mice compared to those treated with AAV9-null vectors (Figure 6A,B). Differences were not observed between the wild-type groups, which died at a very old age (Figure 6A,B).

We next evaluated dopaminergic neurons responsible for motor control and important in Parkinson’s disease by quantifying the tyrosine hydroxylase marker (Figure 6C). We found that G3 *Tert^-/-^* mice treated with AAV9-*Tert* had a higher intensity of tyrosine hydroxylase fluorescence than those treated with the AAV9-null vector (Figure 6C).

Finally, we determined the number of GFAP-positive cells which is indicative of neuroinflammation. We found significantly lower levels of cells positive for GFAP in the hippocampus, dentate gyrus, and neocortex in G3 *Tert^-/-^* mice treated with the AAV9-*Tert* virus compared to the AAV9-null treated controls (Figure 6D,E). Thus, the AAV9-*Tert* therapy shows a trend towards reducing the level of inflammation in the brain, which reaches statistical significance between some of the groups.

In summary, the fact that we observed fewer cells with DNA damage, higher levels of neurogenesis, less inflammation, and more tyrosine hydroxylase in dopaminergic neurons indicates that the AAV9-*Tert* gene therapy had an impact on these molecular markers of aging in the brain.

### AAV9-*Tert* gene therapy has beneficial effects on memory

**Figure 7:**
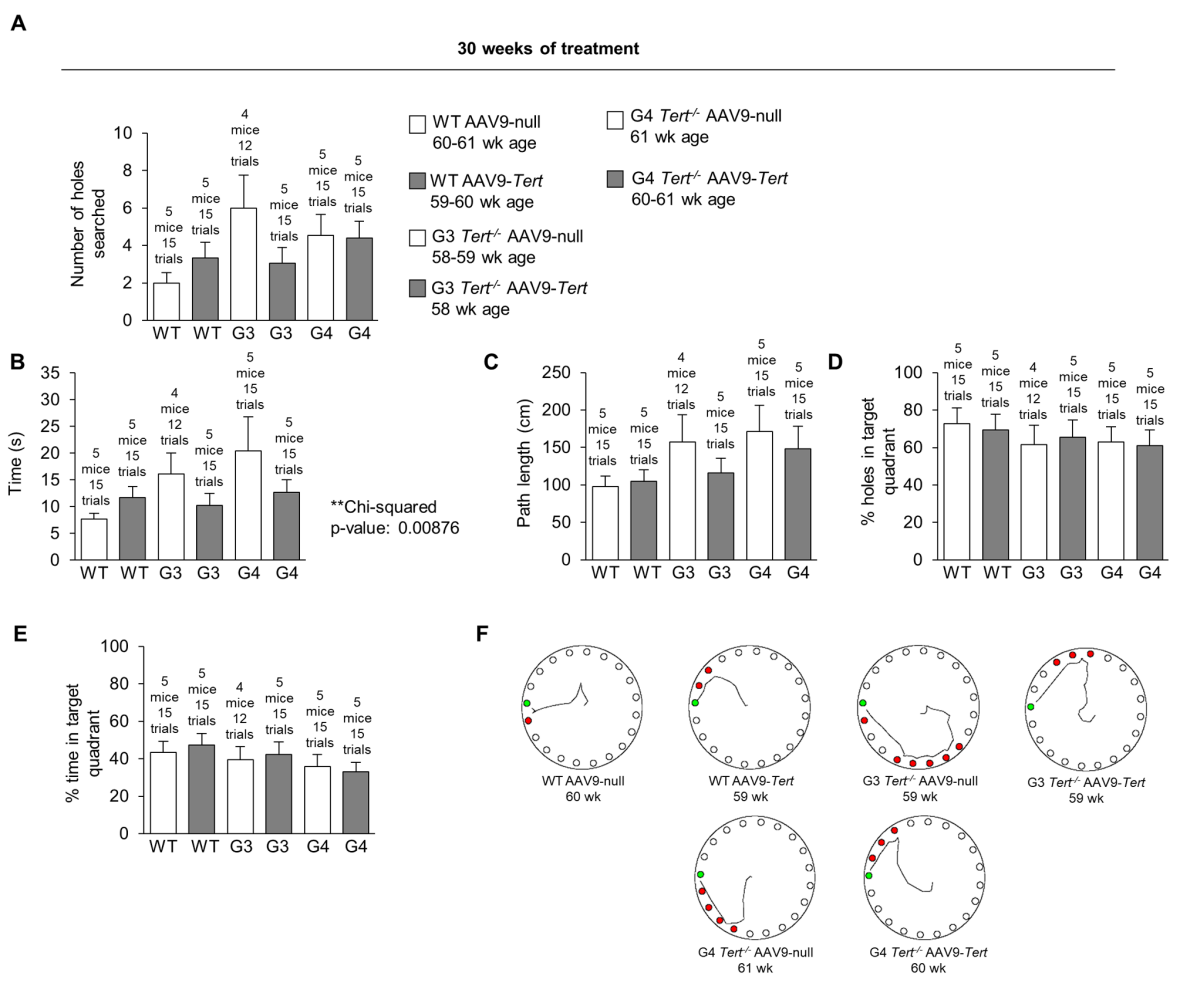
**Treatment with AAV9-*Tert* improves memory in the Barnes maze test**. (**A-E**) Quantification of the performance in the Barnes maze test after 30 weeks of treatment for (**A**) number of holes searched before reaching the goal box, (**B**) time required to reach the goal box, (**C**) path length used to reach the goal box, (**D**) the percent of holes searched in the target quadrant, and (**E**) the percent time spent searching in the target quadrant. (**F**) Representative paths of the mice on the Barnes maze table after 30 weeks of treatment. Data represent the mean ±SE of analyzed mice within each group. The number of mice analyzed per group is indicated.

In order to assess whether telomerase treatment had any impact on brain function, we measured the memory of the mice using the Barnes maze test [90]. In the Barnes maze test, mice are placed onto a table with 20 holes around the edge. Only one of the holes contains a box which the mice can enter. The mice try to escape the aversive stimulus of a wide-open space where they are exposed, and they try to find and enter the goal box for safety. The mice are first guided to the location of the box with a glass beaker on the first habituation day. They are then allowed to find the box on their own for several trials for two training days. After this there is one day of rest, and then the performance of the mice is measured on the final test day. At 30 weeks of treatment, the performance of most of the AAV9-*Tert* groups was better than the performance of the corresponding AAV9-null groups (Figure 7). The number of holes that the mice searched before finding the goal box is shown in Figure 7A. A chi-squared test showed that the mice treated with AAV9-*Tert* required less time to find the goal box hidden beneath one of the 20 holes on the outer edge of the table compared with the corresponding AAV9-null treated controls (Figure 7B). The graphs for the path length used to find the goal box (Figure 7C), the percent of the holes searched in the target quadrant of the table (Figure 7D), and the percent time spent searching in the target quadrant (Figure 7E) are also shown. Additionally, G3 and G4 *Tert^-/-^* mice performed more poorly in this Barnes maze test than the wild-type controls. We also constructed representative paths taken by the mice in different treatment groups (Figure 7F). These paths were obtain-ed from videos of the mice performing the test (Figure 7F). In order to address statistical significance in this test for behavior and memory, a chi-squared test was performed by counting the number of AAV9-*Tert* test trials in which the value was better than the average value for the corresponding AAV9-null group of the same generation. These counts were compared to the number of trials that would be expected to be better by chance if the AAV9-*Tert* therapy had no effect, which is half of the total number of trials (Table 1). The p-value from this chi-squared test for the time variable was significant with a value of 0.00876, in agreement with the fact that nearly all the AAV9-*Tert* trials had a better value than the corresponding AAV9-null group average value, with a few exception trials. A similar chi-squared test also showed that younger AAV9-null mice (approximately 30-week old mice after 4 weeks from the injection with the AAV9-null virus) performed better than older AAV9-null mice (approximately 60-week old mice after 30 weeks from the injection with the AAV9-null virus) as shown in Table 2, supporting the fact that neuro-degeneration naturally occurs with age.

**Table 1:** Barnes maze chi-squared analysis: Comparison of AAV9-*Tert* and AAV9-null mice 30 weeks after treatment.

**Number of holes searched**					
**Group**	**Number of Mice**	**Total trials**	**Trials with performance better than corresponding AAV9-null group average**	**Trials expected by chance**	
WT AAV9-*Tert*	5	15	5	7.5	Chi-squared p-value 0.109
G3 *Tert^-/-^* AAV9-*Tert*	5	14	12	7	
G4 *Tert^-/-^* AAV9-*Tert*	5	15	7	7.5	
					
					
**Time**					
**Group**	**Number of Mice**	**Total trials**	**Trials with performance better than corresponding AAV9-null group average**	**Trials expected by chance**	
WT AAV9-*Tert*	5	15	6	7.5	**Chi-squared p-value 0.00876
G3 *Tert^-/-^* AAV9-*Tert*	5	14	13	7	
G4 *Tert^-/-^* AAV9-*Tert*	5	15	13	7.5	
					
					
**Path length**					
**Group**	**Number of Mice**	**Total trials**	**Trials with performance better than corresponding AAV9-null group average**	**Trials expected by chance**	
WT AAV9-*Tert*	5	15	9	7.5	Chi-squared p-value 0.0951
G3 *Tert^-/-^* AAV9-*Tert*	5	14	12	7	
G4 *Tert^-/-^* AAV9-*Tert*	5	15	10	7.5	
					
					
**Percent holes in target quadrant**					
**Group**	**Number of Mice**	**Total trials**	**Trials with performance better than corresponding AAV9-null group average**	**Trials expected by chance**	
WT AAV9-*Tert*	5	15	3	7.5	Chi-squared p-value 0.237
G3 *Tert^-/-^* AAV9-*Tert*	5	14	6	7	
G4 *Tert^-/-^* AAV9-*Tert*	5	15	7	7.5	
					
					
**Percent time in target quadrant**					
**Group**	**Number of Mice**	**Total trials**	**Trials with performance better than corresponding AAV9-null group average**	**Trials expected by chance**	
WT AAV9-*Tert*	5	15	4	7.5	Chi-squared p-value 0.354
G3 *Tert^-/-^* AAV9-*Tert*	5	14	8	7	
G4 *Tert^-/-^* AAV9-*Tert*	5	15	6	7.5	

Chi-squared tables to calculate statistical significance for the mice after 30 weeks of treatment comparing AAV9-*Tert* treated mice with their corresponding controls of the same treatment age and generation

**Table 2:** Barnes maze chi-squared analysis: comparison of young and old AAV9-null mice 30 weeks after treatment.

**Number of holes searched**					
**Group**	**Number of Mice**	**Total trials**	**Trials with performance better than corresponding AAV9-null group average**	**Trials expected by chance**	
WT AAV9-*Tert*	5	15	12	7.5	Chi-squared p-value 0.0541
G3 *Tert^-/-^* AAV9-*Tert*	4	12	9	6	
G4 *Tert^-/-^* AAV9-*Tert*	5	15	11	7.5	
					
**Time**					
**Group**	**Number of Mice**	**Total trials**	**Trials with performance better than corresponding AAV9-null group average**	**Trials expected by chance**	
WT AAV9-*Tert*	5	15	13	7.5	**Chi-squared p-value 0.00732
G3 *Tert^-/-^* AAV9-*Tert*	4	12	11	6	
G4 *Tert^-/-^* AAV9-*Tert*	5	15	11	7.5	
					
**Path length**					
**Group**	**Number of Mice**	**Total trials**	**Trials with performance better than corresponding AAV9-null group average**	**Trials expected by chance**	
WT AAV9-*Tert*	5	15	12	7.5	*Chi-squared p-value 0.0278
G3 *Tert^-/-^* AAV9-*Tert*	4	12	11	6	
G4 *Tert^-/-^* AAV9-*Tert*	5	15	9	7.5	
					
**Percent holes in target quadrant**					
**Group**	**Number of Mice**	**Total trials**	**Trials with performance better than corresponding AAV9-null group average**	**Trials expected by chance**	
WT AAV9-*Tert*	5	15	12	7.5	Chi-squared p-value 0.0807
G3 *Tert^-/-^* AAV9-*Tert*	4	12	9	6	
G4 *Tert^-/-^* AAV9-*Tert*	5	15	10	7.5	
					
**Percent time in target quadrant**					
**Group**	**Number of Mice**	**Total trials**	**Trials with performance better than corresponding AAV9-null group average**	**Trials expected by chance**	
WT AAV9-*Tert*	5	15	10	7.5	**Chi-squared p-value 0.00851
G3 *Tert^-/-^* AAV9-*Tert*	4	12	12	6	
G4 *Tert^-/-^* AAV9-*Tert*	5	15	12	7.5	

## DISCUSSION

Mouse models of Parkinson’s and Alzheimer’s disease have been generated in the past, however, many of these mouse models have failed to reproduce the complexity of the human pathologies [69,91–93], most likely because these diseases are normally associated with aging, and the aging process may not have been fully modeled in the tested mouse models. One of the hallmarks of aging is the progressive shortening of telomeres, which are the end-capping structures of chromosomes and essential for chromosomal stability [11]. When telomeres reach a critically short length this is sufficient to trigger a persistent DNA damage response, and to trigger cellular senescence and/or apoptosis [94]. Short telomeres also impair the ability of stem cells to regenerate tissues, thus contributing to tissue aging [12,13]. Here, we set to test whether a mouse model of premature aging owing to the presence of short telomeres showed signs of molecular brain aging similar to those that occur with physiological aging. In this regard, we describe here that wild-type mice show signs of neurodegeneration with aging and that these signs are anticipated in the context of the telomerase-deficient mouse model. These findings suggest that telomere shortening may be one of the determinants of brain aging. Future studies warrant analysis of telomere length in other models of Alzheimer’s disease, such as including the APP/PS1 mouse model, expressing mutated amyloid precursor protein (APP) and mutated presenilin (PS1) [95], the SAMP8 (Senescence Accelerated Mouse-Prone 8) mouse model [96], which exhibits features of accelerated aging, signs of neurodegeneration at an early age, and poor performance in memory tests [96], or in mice injected with amyloid beta peptide, which induces cognitive defects [97]. In particular, the increased inflammation often observed in these mouse models, may lead to the proliferative exhaustion of immune cells in the brain such as microglia, and ultimately shorter telomeres. Here, we observed increased neurodegeneration with age in both old wild-type mice and in G3 *Tert^-/-^* mice with short telomeres. Indeed, old age is the main risk factor for many diseases, including neurodegenerative diseases [98–100]. Telomerase-deficient G3 *Tert^-/-^* mice had smaller brains and shorter telomeres in the brain, and this was coincidental with increased brain aging as indicated by a number of molecular markers. In some instances, however, the differences between wild-type and the G3 *Tert^-/-^* mice were lost at old ages. A possible explanation for this finding is that the alteration in these markers already reached the maximum or minimum level at older ages, and therefore a difference between the wild-type and G3 *Tert^-/-^* mice was not observed.

Preventing accumulation of short telomeres may prevent or ameliorate brain aging by allowing stem cells to proliferate and regenerate damaged tissue. We have previously demonstrated that preventing accumulation of short telomeres through telomerase gene therapy can ameliorate the symptoms of cardiovascular disease [25], pulmonary fibrosis [26], aplastic anemia [24], and aging in general [23].

Thus, to demonstrate that telomere shortening may be one of the causes of brain aging, here we studied the potential therapeutic effects of a telomerase gene therapy in ameliorating molecular signs of neurodegeneration associated with physiological mouse aging as well as in the context of the telomerase-deficient mouse model. There have been many successes for gene therapy in the last decade [101–107], and the first gene therapy was licensed for human use in Europe in 2012 (Glybera) [108]. Viral vectors have also been used to transduce a wide variety of tissues from liver, skeletal muscle, heart, brain, lung, pancreas, to tumors [109]. Our findings demonstrate that AAV9-*Tert* treatment can ameliorate signs of neurodegeneration with aging in wild-type mice as well as in the context of the telomerase-deficient mouse model with the presence of short telomeres. Our treatment was applied through an IV tail injection, and therefore, many other cell types throughout the body would be infected in addition to the cells in the brain. Improvements of health in other organs may have an impact on the brain and investigating the nature of this relationship could be interesting for future studies. Note also that we did not observe any increased incidence of cancer in the mice treated with AAV9-Tert, which matched our expectations since several other articles have demonstrated that telomerase reactivation alone does not lead to tumorigenesis in vivo [23,39,110,111].

Of note, the AAV9 serotype used here to express telomerase in the brain primarily transfects neurons and astrocytes but fails to transduce microglia [77]. In our experimental setting, we found that less than 5% of the cells in the brain received the transgene using our vector and delivery method. Interestingly, in spite of the low transduction efficiency, we observed significant effects of AAV9-*Tert* gene therapy in decreasing DNA damage, increasing neurogenesis as indicated by increased doublecortin expression, as well as decreasing neuroinflammation (decreased GFAP expression). These findings suggest that even a small number of neurons transduced with *Ter*t may increase the health of the environment and benefit cells that were not infected, for instance, through changing the secretory profile of cells. In an analogous manner, factors found in young blood induce a younger phenotype in the recipient cells, as observed from parabiosis experiments with young blood [112,113] and also treatments with specific factors in young blood such as the GDF11 protein [114,115]. Nevertheless, even more benefits from telomerase gene therapy may be observed if higher transduction efficiencies are obtained.

## MATERIALS AND METHODS

### Mice

For all the treatment groups, mice were injected IV in the tail with 2E12 vg of AAV9 virus particles containing the genetic material of interest in a volume of 100 μL 0.001% Pluronic F-68 in PBS. The Pluronic F-68 serves as a surfactant to prevent aggregations of the virus particles from forming [77]. In the experiment to confirm infection of mouse brain cells, mice were injected with either AAV9-CMV-*eGFP*, AAV9-CAG-*eGFP*, or AAV9-CMV-*Tert*. In the neurodegeneration aging study, the mice were injected with either AAV9-*Tert* or AAV9-null. The mice were injected at an age of 27-30 weeks. The mice were genetically wild-type or knockout for telomerase. Throughout these studies, the telomerase knockout mice belonged to different generations: either G1, G2, G3, or G4 *Tert^-/-^* genera-tions. The mouse strain was >95% C57BL/6 back-ground, and all of the mice used in these experiments were male.

All mice were produced and housed in the specific pathogen-free barrier of the CNIO institution in Madrid Spain. After weaning, five mice were housed per cage and fed ad libitum with a non-purified sterilizable Teklad 2018 18 percent protein rodent diet (Harlan TD.2018S). All animal procedures were approved by the CNIO-ISCIII Ethics Committee for Research and Animal Welfare (CEIyBA) and conducted in accor-dance to the recommendations of the Federation of European Laboratory Animal Science Associations. 

### MPTP Neurotoxin treatment

Wild-type or G3 *Tert^-/-^* 12-16-week old male mice were injected IP with saline or MPTP neurotoxin at a concentration of 0.5, 5, 10, or 20 mg/kg body weight. Behavior tests such as the footprint test and tail suspension test were then performed. The mice were sacrificed in a CO_2_ chamber 7 days after injection, and the brain was preserved in formalin for subsequent molecular tests.

### Recombinant AAV vectors

Vectors were generated by triple transfection of HEK293 cells as described previously [116]. Cells were cultured in roller bottles (Corning) in DMEM 10% FBS to 80% confluence and co-transfected with a plasmid carrying the expression cassette flanked by the AAV2 viral ITRs, a helper plasmid carrying the AAV rep2 and cap9 genes, and a plasmid carrying the adenovirus helper functions (kindly provided by K.A. High, Children’s Hospital of Philadelphia). The expression cassettes used were one of the following: *eGFP* under the control of the CMV promoter, *eGFP* under the control of the CAG promoter, or murine *Tert* under the control of the CMV promoter. AAV9 vectors were purified with an optimized method based on two consecutive cesium chloride gradients, dialyzed against PBS, filtered, and stored at -80 °C until use [117]. The titer of the viral genome particles was determined by quantitative real time PCR.

### Confirmation of virus infection and measurement of transduction efficiency

Groups of 8-week old mice were IV tail injected with 2E12 vg of AAV9 vectors containing different pieces of genetic material in 100 μL of 0.001% Pluronic F-68 in PBS. The mouse group size and identity of the injected vectors were as follows: 3 mice AAV9-CMV-*Tert*, 3 mice AAV9-CMV-*eGFP*, 2 mice AAV9-CAG-*eGFP*, and 3 control mice receiving no vector. At 2 weeks post-injection, the mice were sacrificed, and IVIS was used to measure the fluorescence in the AAV9-CMV-*eGFP* mice and controls. Then half the brain was preserved in liquid nitrogen for freezing, and half the brain was preserved in formalin for paraffin sections. RNA was extracted from frozen tissue, and a qPCR was performed to check the level of the *Tert* and *eGFP* expression. The paraffin sections were used in an immunohistochemistry test for GFP, and for an immunofluorescence experiment for GFP and the microglia marker Iba1. The GFP antibody used in immunohistochemistry was mouse monoclonal anti-GFP (Roche Cat No 11 814 460 001) as described in more detail in the immunohistochemistry section. The GFP antibody used in immunofluorescence was chicken anti-GFP (Aves Labs Inc. Cat No GFP-1020), and the Iba1 antibody used was rabbit anti-Iba1 (Wako Cat No 019-19741).

### Neurodegeneration aging experiment

To assess whether AAV9-*Tert* gene therapy could reduce neurodegeneration with age, mice were injected with either AAV9-null or AAV9-*Tert* virus. The mice were injected IV in the tail with 2E12 vg of virus in a volume of 100 μL 0.001% Pluronic F-68 in PBS. The mice were injected at an age of 27-30 weeks. The cohort consisted of the following mice: 5 wild-type AAV9-null, 5 wild-type AAV9-*Tert*, 4 G3 *Tert^-/-^* AAV9-null, 5 G3 *Tert^-/-^* AAV9-*Tert*, 5 G4 *Tert^-/-^* AAV9-null, and 5 G4 *Tert^-/-^*AAV9-*Tert* mice. After the injection, all the mice were allowed to age until the humane endpoint. Behavioral and cognitive tests such as the Barnes maze test, object recognition test, rotarod test, tightrope test, and tail suspension test were performed at various timepoints throughout the lifespan of the mice. At the humane endpoint, the mice were sacrificed in a CO_2_ chamber. Half of the brain was preserved in nitrogen for freezing, and half the brain was preserved in formalin for paraffin sections. The paraffin sections cut in a coronal orientation were used for q-FISH to measure telomere length, and for immunohistochemistry to measure the markers γH2AX, doublecortin, and GFAP.

### Telomere Q-FISH analysis of paraffin sections

Paraffin-embedded tissue sections were hybridized with a PNA-telomeric Cy3 probe, and fluorescence intensity of telomeres was determined as described previously [118,119]. After deparaffinization, tissues were post fixed in 4% formaldehyde for 5 min, washed 3X5 min in PBS and incubated at 37 °C for 15 min in pepsin solution (0.1% Porcine Pepsin, Sigma; 0.01 M HCl, Merck). After repeating another round of washes and fixation, slides were dehydrated in a 70%–90%–100% ethanol series (5 min each). After air drying the slides, 30 μL of telomere probe mix (10 mM TrisCl pH 7, 25 mM MgCl_2_, 9 mM citric acid, 82 mM Na_2_HPO_4_, 70% deionized formamide (Sigma), 0.25% blocking reagent (Roche) and 0.5 μg/mL Telomeric PNA probe (Panagene)) were added to each slide. A cover slip was applied, and the slides were incubated for 3 min at 85 °C. The slides were then incubated 2 hr at room temperature in a wet chamber in the dark. Slides were washed 2X15 min in 10 mM TrisCl pH 7, 0.1% BSA in 70% formamide with shaking, then 3X5 min in TBS 0.08% Tween 20, and then incubated in a 4 μg/mL 4',6-diamidino-2-phenylindole DAPI (Sigma) bath in PBS before mounting samples in Vectashield (VectorTM). Confocal images were acquired as stacks using an SP5-WLL confocal microscope (Leica) and maximum projection images were created using the Fiji version of the ImageJ software (NIH) [120,121]. Telomere signal intensity was quantified using Definiens Developer Cell software (version XD 64 2.5; Definiens AG).

### Immunohistochemistry

Brains were fixed in 10% neutral buffered formalin with 4% formaldehyde, paraffin-embedded, cut at 3 µm, mounted in Superfrost Plus slides (Thermo Fisher Scientific), and dried overnight. The paraffin brain sections were cut in a coronal orientation and a series of sections from the posterior part of the brain to the anterior part of the brain were placed onto a slide. For different staining methods, slides were deparaffinized in xylene and re-hydrated through a series of graded ethanol washes until a final wash with water. Consecutive sections were stained with an automated Ventana Discovery XT immunostaining platform (Roche). Antigen retrieval was first performed with high pH CC1m buffer (Roche) and endogenous peroxidase was blocked with 3% hydrogen peroxide. Next the slides were incubated with one of the following primary antibodies: pre-diluted rabbit monoclonal SP6 anti-Ki67 (Master Diagnostica Cat No 000311OQD), mouse monoclonal JBV301 anti-γH2AX at a 1/25,000 dilution (Millipore Cat No 05-636), mouse monoclonal 7.1+13.1 anti-GFP at a 1/500 dilution (Roche Cat No 11.814.460.001), rabbit polyclonal anti-doublecortin at a 1/500 dilution (Abcam Cat No ab18723), rabbit polyclonal anti-glial fibrillary acidic protein (GFAP) at a 1/750 dilution (DAKO Z0334), or rabbit monoclonal anti-tau (phospho S396) at a 1/2000 dilution (Abcam Cat No ab10930). After the primary antibody step, slides were incubated with either the rabbit anti-mouse secondary antibody (Epitomics) or the OmniMap anti-rabbit detection system (Ventana Roche) conjugated with horseradish peroxidase. The immunohistochemistry (IHC) reaction was developed using ChromoMap 3,3'-Diaminobenzidine tetrahydrochloride DAB (Ventana Roche) and nuclei were counterstained with Carazzi’s hematoxylin. Finally, the slides were dehydrated, cleared, and mounted with a permanent mounting medium for microscopic evaluation. Positive control sections known to be positive for the primary antibody were included for each staining procedure. Images of whole slides were acquired with an AxioScan Z1 slide scanner (Zeiss) and visually checked with the Zen Blue software (Zeiss). The digital images from the scan were then quantified by counting the number of positive cells in different fields of view. Either Zen Blue software (Zeiss) or the Fiji version of the ImageJ software (NIH) [120,121] was used for this quantification.

### Immunofluorescence

For immunofluorescence (IF), the slides were first prepared and antigen retrieval was performed as described previously in the immunohistochemistry section. Next the slides were washed 2X5 min in PBS. The slides were then permeabilized in 0.5% Triton X-100 in PBS for 3 hr at room temperature (RT), washed 5 min in 1X PBS, blocked with fetal bovine serum (FBS) for 2 hr, and then the slides were incubated with primary antibody overnight at 4 °C in a humidity chamber. The primary antibodies used were one of the following: rabbit polyclonal anti-tyrosine hydroxylase (Millipore Cat No AB152), chicken anti-GFP (Aves Labs Inc. Cat No GFP-1020), or rabbit anti-Iba1 (Wako Cat No 019-19741). Next the slides were washed 4X7 min with 1XPBS 0.1% Tween 20 at RT, washed with 1XPBS 5 min, and incubated with secondary antibody 1 hr at RT in a humidity chamber. The secondary antibodies used were one of the following: goat anti-rabbit Cy3 (Jackson Cat No 4134) or goat anti-chicken AF488 (Life Technologies Cat No A11039). Next the slides were washed 3X10 min with PBS, incubated with DAPI 5 min at RT, washed 5 min with PBS, 30 μL of Vectashield (VectorTM) was added, and the slides were sealed with nail polish. Confocal images were acquired as stacks using a SP5-WLL confocal an microscope (Leica) and maximum projection images were created using the Fiji version of the ImageJ software (NIH) [120,121]. Fluorescence intensity was quantified using Definiens Developer Cell software (version XD 64 2.5; Definiens AG).

### Quantitative real-time PCR

Total RNA from nitrogen frozen tissues was extracted with Qiagen’s RNeasy mini kit (Qiagen Cat No 74106), according to the manufacturer’s instructions. Before processing, RNA samples were DNaseI treated using the RNase-free DNase Set (Qiagen Cat No 79254) according to the manufacturer’s instructions. First-strand cDNA was synthesized from this RNA using an iScript cDNA synthesis kit (Bio-RAD Laboratories, Inc. Cat No 1708891) according to the manufacturer’s instructions. The reaction consisted of 15 μL of 1 μg RNA, 4 μL of 5X iScript reaction mix, and 1 μL iScript reverse transcriptase. A Primus 96 Plus thermocycle instrument was used with the following settings: 5 min 25 °C; 30 min 42 °C; 5 min 85 °C; 4 °C hold. The PCR reactions were performed using the GoTaq qPCR Master Mix (Promega Cat No A6002) according to the manufacturer’s instructions. The reaction mix consisted of 6 μL GoTaq PCR master mix, 0.2 μL 10 uM forward primer, 0.2 μL 10 μM reverse primer, 2 μL of 1/10 dilution of first-strand cDNA, and 3.6 μL H20. Quantitative real-time PCR was performed for these reactions using an ABI PRISM 7700 384 well plate reader (Applied Biosystems) according to the manufacturer’s instructions. The thermocycle program was as follows: Hold stage (1.6 °C/s ramp; 50 °C 2 min; 1.6 °C/s ramp; 95 °C 10 min); PCR Stage with 40 cycles (95 °C 0:15 min; 1.6 °C/s ramp; 60 °C 1 min; 1.6 °C/s ramp; 68 °C 1 min); Melt Curve Stage (1.6 °C/s ramp; 95 °C 0:15 min; 1.6 °C/s ramp; 60 °C 1 min; 0.05 °C/s ramp; 95 °C 0:15 min). The following primers were used for PCR reactions: TERTFor: 5’-GGATTGCCACTGGCTCCG-3’, TERTRev: 5’-TGCCTGACCTCCTCTTGTGAC-3’, GFPFor: 5’-ACCCTGAAGTTCATCTGCA-3’, GFPRev: 5’-GGACTTGAAGAAGTCGTGC-3’, Trp53For: 5’-GTCACAGCACATGACGGAGG-3’, Trp53Rev: 5’-TCTTCCAGATGCTCGGGATAC-3’, mActFor: 5’-GGCTGTATTCCCCTCCATCG-3’, mActRev: 5’-CCAGTTGGTAACAATGCCATGT-3’, GAPDHFor: 5’-AATGGCAGCCCTGGTGAC-3’, GAPDHRev: 5’-AGACGGCCGCATCTTCTT-3’.

### Micro PET imaging

Images were acquired using an eXplore Vista PET-CT instrument (GE Healthcare). Mice were injected with 15 MBq (megabecquerel) of 18F-FDG (ITP Cyclotron) into the lateral tail vein in a volume of 0.1 cc. During imaging, mice were anaesthetized with a continuous flow of 1% to 3% isoflurane/oxygen mixture (2 L/min) 45 min after radiotracer injection. MicroPET scans were performed for 20 min per bed. Only one mouse was used per bed. PET images were reconstructed with a three-dimensional Ordered Subsets Expectation-Maximization (OSEM) reconstruction algorithm and were analyzed using MMWS software (eXplore Vista, GEHC) [122]. The images were analyzed by drawing a region of interest (ROI) and calculating the 18F-FDG SUV (standardized uptake value) using the following formula: SUV = Tissue concentration ROI activity (Mbq/ml) / (Injected dose (Mbq) / Body weight (g)).

### IVIS

The fluorescence intensity of the mice was measured immediately after sacrifice in a CO_2_ chamber. An IVIS Spectrum (Xenogen) instrument was used to measure fluorescence, and the Living Image version 4.3.1 (Caliper Life Sciences, Inc.) software was used for quantification. The fluorescence intensity was dis-played with log scale and rainbow coloring with a min value of 511 and a max value of 1205. The fluorescence intensity of the desired region of interest (ROI) was then quantified.

### Behavioral and cognitive tests

Several behavioral and cognitive tests were performed with the mice to measure health. The rotarod test was used to evaluate motor coordination and balance in mice [123]. The mice were tested in a rotarod apparatus (Panlab model LE 8200) using a continuous acceleration protocol from 4 to 40 rpm in a period of 120 s. The time before the mice fell was recorded, and the average of three trials was used in the quantification. In the tightrope test [124,125], the mice were placed onto a rod (“the tightrope”) for 5 trials for 60 s. Each trial that the mouse did not fall was counted as a success, and the percent success for each mouse was determined.

The object recognition test measures the ability of mice to remember objects in their environment and provides an index of recognition memory [72,126]. The mice were first presented with two identical objects for 10 min. Then after 24 hours, the mice were presented with one object that was identical to the previous objects and one novel object. The amount of time the mouse spent investigating the new object by sniffing or touching the new object indicated that the mouse may have recognized that this object was new. The final results were presented as the ratio of time the mouse spent investigating the new object to the total time investigating both objects.

The tail suspension test evaluates psychological depression, motivation, and energy levels in mice by measuring the amount of time the mice spend trying to escape by struggling and moving when hung from their tail [70]. Mice typically struggle less and move less with old age. Video recordings were captured for each mouse, and the amount of movement was then quantified. More movement indicates less depression, more motivation, and higher energy levels. The mice were hung by the tail using a length of tape of approximately 17 cm. A plastic tube cut from a 10 mL pipette was placed around the tail before attaching the tape in order to prevent the mice from climbing their own tails during the test. Videos of the mice were captured using a standard PowerShot SX430 camera (Canon) held stable by a STAR 75 tripod (Hama). Analysis of the video was performed by first converting the video into a sequence of images using the VLC VideoLAN Client (VideoLAN Organization) [127]. This image sequence was then analyzed with the Fiji version of the ImageJ software (NIH) [120,121]. Briefly, the color was inverted so that the white tape became black and the black background became white, a z-projection of the average intensity was created to identify where the mouse was located the majority of the time throughout the video sequence, a rectangular region of interest (ROI) was created around the region of tape close to the mouse, the ROI was restored onto the original image sequence (instead of the single frame z-projection), the integrated intensity of the ROI in each image was measured and output to a spreadsheet, and the number of image frames in which the integrated density was greater than the threshold was determined. These frames indicate that the mouse was moving since more white background is detected in these frames instead of the black tape which would be in the frame if the mouse was not moving. The amount of time that the mouse was immobile was then displayed in a bar graph. This method of analysis correctly yielded long immobility times for mice which moved very little, and short immobility times for mice that were moving frequently.

The footprint test measures the stride length of the mice and has been used to quantify mobility in Parkinson’s-like mouse models [128,129]. The paws of the mice were painted with watercolor paint. The front and hind limbs were painted with a different color. The mice were then placed into a hallway with paper on the floor. The hallway was 8 cm wide and 60 cm long. The mice then moved through the hallway into a dark container at the end which they could hide in. Three trials were performed with each mouse, and the distance between the footprint paint marks on the paper was then measured with a ruler. The hind paw stride length was the average of the lengths for both the right and left paws, and the front paw stride length was measured in the same manner.

The Barnes maze test is used to evaluate the learning ability and spatial memory of mice [130–132]. The mice are trained to find a hole leading to an escape box on a table with many other holes. Visual cues such as simple colored shapes around the room and normal objects in the room were used so that the mice could orient themselves on the table. The training consisted of a habituation day in which the mice were guided to the target box with a glass beaker from the center of the table, the first training day in which the mice were allowed to explore the table for 3 trials for 2 min, a second training day in which the mice were allowed to explore the table for 2 trials for 2 min, a resting day, and then the final test day in which 3 trials of 2 min were performed. Aversive stimuli such as a bright light above the table from a flashlight and a digital metronome (Seiko Cat No DM50SE) which makes a repetitive loud noise set at 132 bpm were used to motivate the mouse to search for and escape into the goal box. The wide-open space on the table also serves as an aversive stimulus since the mice are exposed to any threat. Note that the Barnes maze test is often considered to be more humane than the Morris water maze. The final output of the test was represented as the time the mice took to find the target box, the path length, the number of holes searched, the number of holes searched in the target quadrant of the table, and the time spent in the target quadrant of the table. Performance on this test is related to the health of the hippocampus which is responsible for spatial memory.

Videos of the Barnes maze test trials were analyzed to quantify the results. The maintenance facility of the CNIO constructed a goal post-like structure, and a standard PowerShot SX430 camera (Canon) was attached to the top of this structure with a screw above the table to video record the tests. To determine the time required for each mouse to find the goal box, and to determine the hole ids visited before finding the goal box, the videos were simply watched, and the in-formation was recorded. Note that the time recorded refers to the time for the mouse to first find the goal box hole (approach the hole and look into it), not actually enter the hole into the goal box. Sometimes the mice would find the hole, and then wait a considerable amount of time before actually entering the hole. The videos were watched with the SMPlayer media player (https://www.smplayer.info/) so that we could play the videos forward or backward frame by frame and at custom speeds such as twice the speed to assist with the analysis. The SMPlayer window was also made transparent and placed over a diagram of the table with ID numbers for each hole made in the Inkscape vector graphics editor (https://inkscape.org). To determine other variables such as path length, and the percent time in the target quadrant, each video was first converted into an image sequence using the VLC VideoLAN Client (VideoLAN Organization) [127]. The image sequence was imported into the Fiji version of the ImageJ software (NIH) [120,121], and analyzed. Briefly, regions of interest (ROIs) for the quadrants were created. Any part of the image scene which was very dark was cleared so that the black mouse was the darkest object in the image. The min value in each quadrant for each frame was then measured. Frames with a min value below a certain threshold were frames which contained the mouse, and the percent time the mouse spent in each quadrant was determined. To quantify the path length, the brightness of the image was increased to the max value, the contrast was increased until most of the artifacts in the image disappeared and only the mouse could be seen. A thres-hold was applied to the image so that only pure white and black colors remained. Then the MTrack2 plugin [133] for Fiji was used to track the movement of the mouse, quantify the path length, and also produce a trace image of the path taken by the mouse. The parameters necessary for the plugin would vary depending on the conditions of the video, but in our videos the following parameters usually produced correct path tracings: Min Particle Size: 300, Max Particle Size: 10,000, Max Speed: 10,000, and Minimum Track Length (frames): 5. The resulting variables of "number of holes searched", "path length", "time", "percent time in target quadrant", and "percent holes in target quadrant" were then graphed.

### Statistical and mathematical analyses

test, tail suspension test, object recognition test, and PET test results since each individual mouse cannot belong to more than one group and the variables are assumed to be normally distributed. A χ^2^ (chi-squared) test was used for the Barnes maze test to determine whether AAV9-*Tert* treated groups performed better than their corresponding AAV9-null controls with a matching generation. This test was implemented since we were comparing categorical variables, specifically whether a mouse performed better or worse than the AAV9-null group of the same generation. For statis-tical tests and mathematical analysis, we used the Excel 2016 software (Microsoft).

## Supplementary Material

Supplementary Figures
