# Supplementary material for: Telomerase gene therapy ameliorates the effects of neurodegeneration associated to short telomeres in mice
Source: Aging (Albany NY). 2019 May 28;11(10):2916–48. doi: 10.18632/aging.101982 (PMC6555470; doi:10.18632/aging.101982)
Supplement: Supplementary Figures [file aging-11-101982-s001.pdf]

SUPPLEMENTARY FIGURES

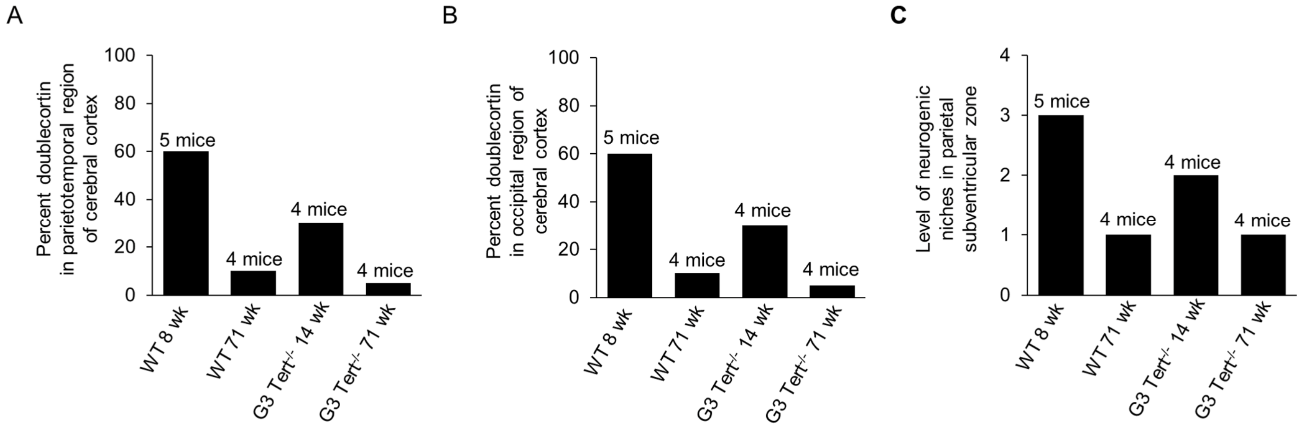

Figure S1. Younger mice and wild-type mice have more doublecortin in the cerebral cortex than older and G3 *Tert*$^{-/-}$ mice and more neurogenic niches in the parietal subventricular zone. (**A**) Percent doublecortin in parietotemporal region of cerebral cortex. (**B**) Percent doublecortin in occipital region of cerebral cortex. (**C**) The level of neurogenic niches in the parietal subventricular zone quantified from hematoxylin and eosin stained paraffin sections.

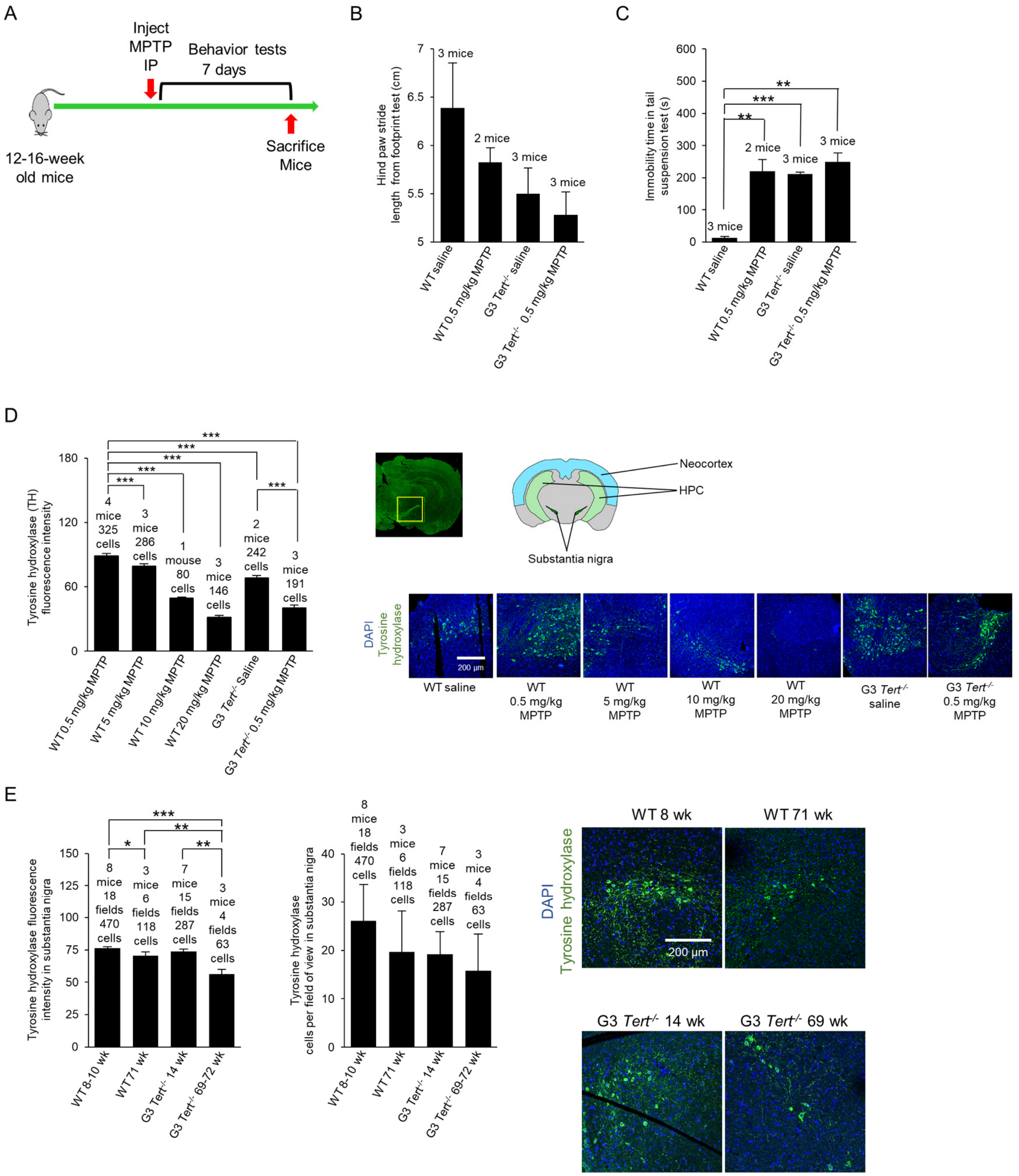

Figure S2. Mice deficient for telomerase are more susceptible to Parkinson's-like symptoms induced by MPTP neurotoxin. (**A**) Scheme of the MPTP neurotoxin experiment. Wild-type or G3 *Tert*$^{-/-}$ mice, were injected with different doses of MPTP or saline solution. Behavior tests were performed, and the mice were sacrificed 7 days after injection. The brain was preserved in formalin. (**B**) Hind paw stride length from footprint test. (**C**) The immobility time in the tail suspension test. (**D**) Immunofluorescence results for tyrosine hydroxylase in the substantia nigra for mice treated with different doses of MPTP neurotoxin. (**E**) Immuno-fluorescence results for tyrosine hydroxylase in the substantia nigra for young and old wild-type and G3 *Tert*$^{-/-}$ mice. Data represent the mean ±SE of analyzed mice within each group. For the histopathology results, the number of mice analyzed per group is indicated, as well as the number of fields of view, and the number of positive cells. The *t*-test was used for statistical analysis. *p<0.05; **p<0.01; ***p<0.001.

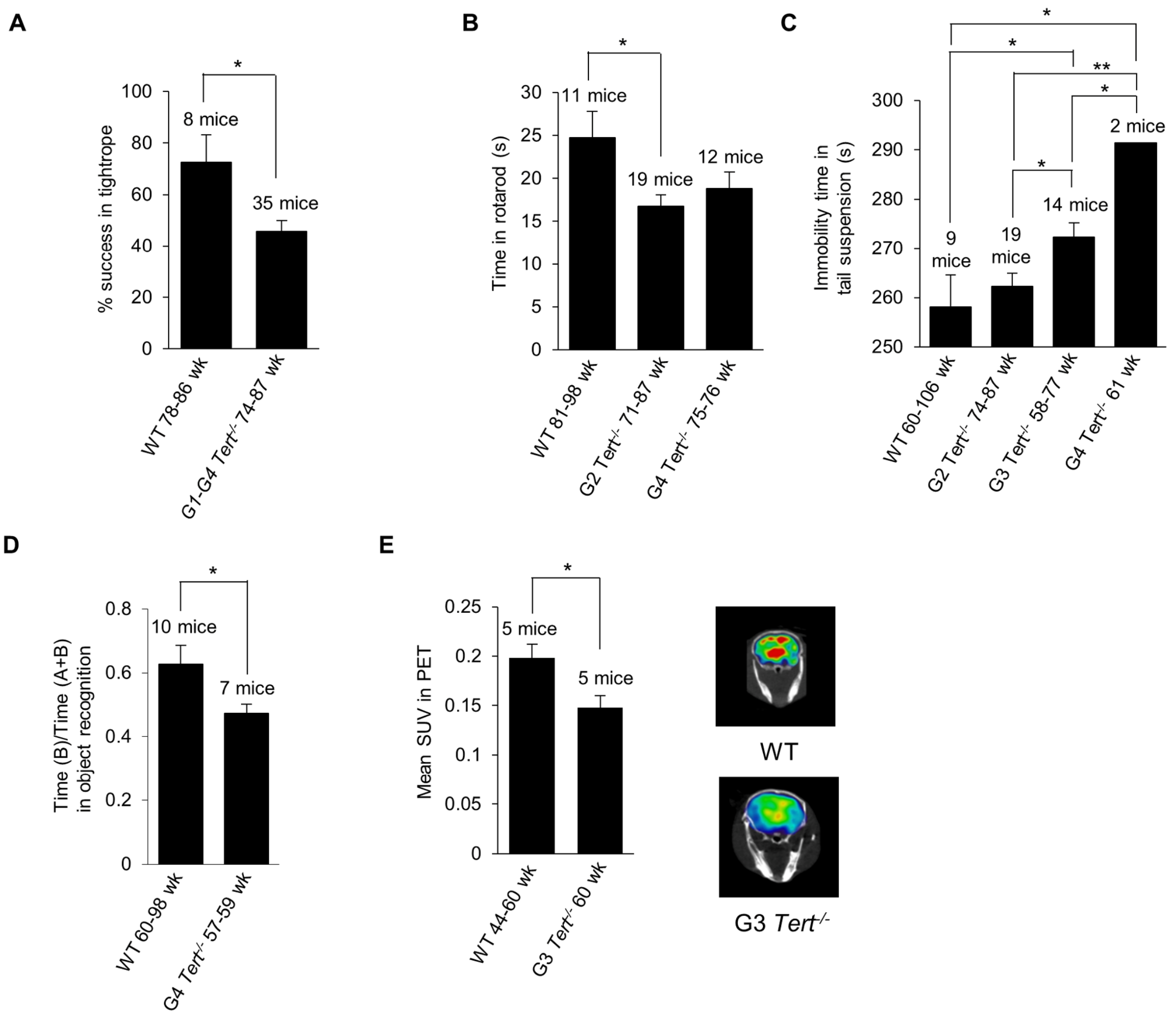

Figure S3. Mice deficient for telomerase perform more poorly in the tightrope test, rotarod test, tail suspension test, and object recognition test, and they also have less glucose uptake in the brain. (**A**) Percent success in the tightrope test for wild-type and *Tert*$^{-/-}$ mice at 70-90 weeks of age. (**B**) Time in rotarod test for wild-type, G2 *Tert*$^{-/-}$, and G4 *Tert*$^{-/-}$ mice at 70-100 weeks of age. (**C**) Immobility time in tail suspension test for old wild-type, G2 *Tert*$^{-/-}$, G3 *Tert*$^{-/-}$, and G4 *Tert*$^{-/-}$ mice. (**D**) Ratio of time spent investigating a novel object (Time B) to the time spent investigating both objects (Time A+B) in the object recognition test. (**E**) Standard uptake value (SUV) of 18F-FDG (18F-fluorodeoxyglucose) into the brain in a positron emission tomography (PET) test. Representative heatmaps displaying the quantity of glucose uptake are also shown. Data represent the mean ±SE of analyzed mice within each group. The number of mice analyzed per group is indicated. The *t*-test was used for statistical analysis. *p<0.05; **p<0.01.

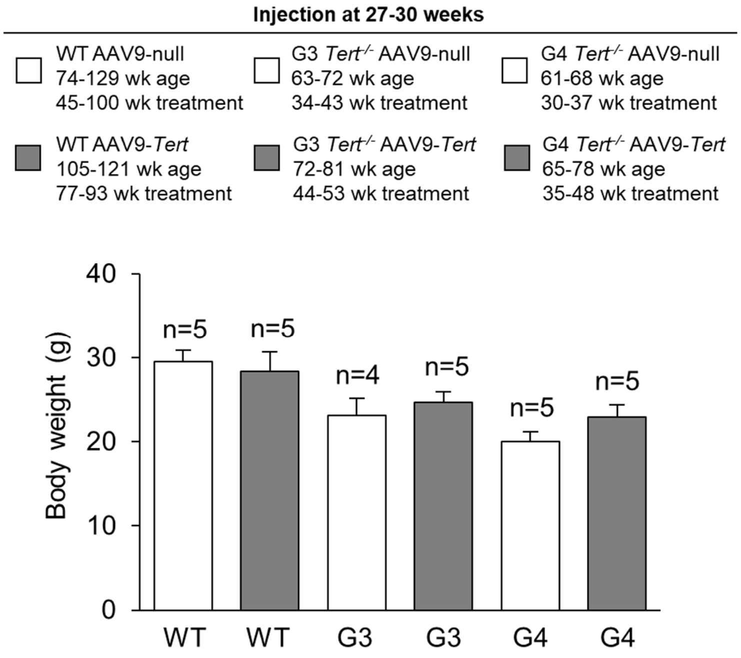

Figure S4. Body weights of the mice treated with AAV9-*Tert* or AAV9-null. These weights were determined at the humane endpoint. The age of death, the weeks of treatment, and the generation for each group is presented.
